# Efficacy and safety of Wenxin Keli combined with metoprolol tartrate in the treatment of premature ventricular contractions: A systematic review and meta-analysis

**DOI:** 10.3389/fcvm.2022.952657

**Published:** 2022-07-29

**Authors:** Ping Huang, Yining Luo, Jiaxue Chen, Jingke Xu, Yuanshu Shi, Guoren Chen, Ping Ma

**Affiliations:** School of Basic Medicine, Chengdu University of Traditional Chinese Medicine, Chengdu, China

**Keywords:** Wenxin Keli, metoprolol tartrate, Ventricular Premature Complexes, meta-analysis, safety, efficacy

## Abstract

**Background:**

Wenxin Keli (WXKL) has good clinical value in the treatment of premature ventricular contractions, but there is insufficient evidence to support it. This study evaluates the efficacy and safety of WXKL combined with metoprolol tartrate in the treatment of ventricular premature beats (VPCs).

**Methods:**

We searched seven databases to identify randomized controlled trials (RCTs) for this study. Two reviewers independently screened and extracted the data. The Cochrane Manual criteria were used for methodological quality assessment. Meta-analyses were performed using Review Manager 5.4.1 software. Risk ratios (RR) were used for effect sizes for dichotomous data, demonstrated in effect sizes and 95% confidence intervals (CIs).

**Results:**

A total of 11 RCTs of WXKL combined with metoprolol tartrate in the treatment of premature ventricular contractions were included in this study. Meta-analysis showed that WXKL combined with metoprolol tartrate (treatment group) was more effective than metoprolol tartrate (control group) in improving premature ventricular contractions (RR = 1.32, 95% CI: [1.24, 1.40], *P* < 0.00001); significantly improved the rate of premature ventricular contractions (RR = 1.32, 95% CI: [1.23, 1.41], *P* < 0.00001); there was no difference in adverse drug reactions compared with the control group (RR = 0.61, 95% CI: [0.35, 0.1.05], *P* = 0.08), but the number of adverse reactions (*n* = 18) was less than that of the control group (*n* = 32), and the severity was lower than that of the control group. The included studies only mentioned randomization and did not describe the generation of random sequences in detail.

**Conclusion:**

This study found that Wenxin Keli combined with metoprolol tartrate in the treatment of premature ventricular contractions increased the efficacy of the drug, reduced the occurrence of adverse reactions, and reduced the severity of adverse reactions. Due to the quality limitations of the included studies, more high-quality RCTs are needed in the future to provide more evidence for longer-term analyses.

## Introduction

Premature ventricular contraction, also known as premature ventricular contraction (PVC), refers to the premature ventricular contraction caused by the premature depolarization of the ectopic excitatory foci of the ventricular muscle below the bundle of His and its branches (1). In the general population, the incidence of premature ventricular contractions is 1 to 4% (2). The prevalence of premature ventricular contraction was found to be approximately 1% by ordinary ECG screening and as high as 40 to 75% by 24-h or 48-h dynamic ECG detection (3). Premature ventricular contractions are a common arrhythmia that affects the pumping function of the heart (4, 5). The clinical manifestations are often chest tightness, palpitations, fatigue, dizziness, and other uncomfortable symptoms and even evolve into fatal rapid ventricular arrhythmia, which can induce or aggravate adverse cardiovascular events such as angina pectoris and heart failure (6). Its ECG is characterized by an early-onset QRS complex with an abnormal shape and long duration (usually >129 ms). Several population-based studies have shown that a higher frequency of VPC is associated with impaired cardiac function and reduces the quality of life and increases the risk of heart failure (HF), cardiomyopathy, or mortality (7, 8).

There is still no large-scale randomized controlled study to verify the efficacy of drugs on premature ventricular contractions without structural heart disease, and for such patients, health education and comfort should be given first (9). If the patient's symptoms cannot be effectively controlled, drug treatment should be considered, but beta-blockers or non-dihydropyridine calcium antagonists have limited efficacy, and only 10 to 15% of patients have premature ventricular suppression >90% (10), and there was no difference compared to placebo (11). It is worth noting that there is less evidence for the use of calcium antagonists than beta-blockers, and these drugs themselves may cause significant arrhythmic symptoms. Although membrane-active antiarrhythmic drugs may be more effective, the risk-benefit ratio of these drugs in patients without structural heart disease is unclear. Although these drugs can improve discomfort in symptomatic patients, in addition to amiodarone, these drugs may increase mortality in patients with severe structural heart disease and premature ventricular contractions and should be carefully evaluated before treatment (10, 12). Radiofrequency ablation is recommended for some patients with structural heart disease and premature ventricular contractions. Radiofrequency ablation is effective and safe, and the complication rate is mostly <1% (9). However, there is still no consensus on when to consider the use of radiofrequency ablation for premature ventricular contractions (1). Moreover, the success rate of radiofrequency ablation is highly correlated with the origin, and the curative effect is unstable. In addition, patients with frequent premature beats are often in an anxious mood, which increases the release of catecholamines and makes premature beats more frequent (13). Drug treatment for clinical PVCs needs to fully consider the therapeutic effects and adverse reactions of drugs, and treatment costs need to be considered for radiofrequency ablation. Therefore, it is an urgent clinical problem to safely and effectively relieve the symptoms of PVC patients, reduce the number of premature ventricular contractions and delay complications.

Wenxin Keli (WXKL), a proprietary Chinese medicine, is the first Chinese-developed antiarrhythmic drug approved by the China Food and Drug Administration and is increasingly used in the treatment of cardiovascular diseases (CVDs) in China (14). WXKL is composed of five main components: Codonopsis Radix (Dang Shen), Polygonati Rhizoma (Huang Jing), Notoginseng Radix Et Rhizoma (San Qi), Ambrum (Hu Po), and Nardostachyos Radix Et Rhizoma (Gan Song). Its effects are replenishing qi and nourishing yin, promoting blood circulation and removing blood stasis, regulating qi, and relieving pain. At present, studies have shown that WXKL can be used to treat atrial fibrillation, ventricular arrhythmia, myocardial infarction, etc. (15–17). The combination of WXKL and metoprolol tartrate can have a synergistic effect on platelet aggregation. Inhibition can improve blood circulation in patients, reduce blood viscosity, and avoid thrombosis (18–21). Although a large number of studies have shown that WXKL has a good effect on patients with premature ventricular contractions, the evaluation of the efficacy and safety of WXKL combined with metoprolol tartrate in the treatment of premature ventricular contractions is still insufficient. Therefore, in this work, we conducted an inductive analysis of the relevant RCT clinical data to evaluate the effects of WXKL on cardioprotection and antiarrhythmic events, providing a basis for researchers to further explore the prevention of WXKL on cardiovascular and cerebrovascular events. Develop new antiarrhythmic drugs based on WXKL and provide some clues for safer and more effective clinical treatment of VPCs.

## Materials and methods

The protocol for this study has been registered in the International Systematic Prospective Register (PROSPERO, 2022 CRD42022329403, Available from: https://www.crd.york.ac.uk/prospero/display_record.php?ID=CRD42022329403).

### Search methods for the identification of studies

We searched the following seven databases: The Cochrane Library, PubMed, Embase, Web of Science, China National Knowledge Infrastructure (CNKI), Wanfang Database, and Chinese Scientific Journal Database (VIP). To collect randomized controlled trials of WXKL combined with metoprolol tartrate in the treatment of premature ventricular contractions. The search time range is limited from the establishment of the database to April 2022. Use the subject word combined with a free word as the search method. Search terms included “wenxin keli,” “Metoprolol,” and “Ventricular Premature Complexes.” The search strategy for the PubMed database is presented in Table 1.

**Table 1:** Pubmed.

**Search number**	**Query**
#1	“Ventricular Premature Complexes” [Mesh]
#2	(Premature Ventricular Beats[Title/Abstract]) OR (Premature Ventricular Beat[Title/Abstract]) OR (Ventricular Beat, Premature[Title/Abstract]) OR (Ventricular Beats, Premature[Title/Abstract]) OR (Ventricular Premature Complex[Title/Abstract]) OR (Premature Ventricular Contractions[Title/Abstract]) OR (Premature Ventricular Contraction[Title/Abstract]) OR (Ventricular Contraction, Premature[Title/Abstract]) OR (Ventricular Contractions, Premature[Title/Abstract]) OR (Ventricular Ectopic Beats[Title/Abstract]) OR (Ectopic Beat, Ventricular[Title/Abstract]) OR (Ectopic Beats, Ventricular[Title/Abstract]) OR (Ventricular Ectopic Beat[Title/Abstract]) OR (Extrasystole, Ventricular[Title/Abstract]) OR (Ventricular Extrasystole[Title/Abstract]) OR (Ventricular Extrasystoles[Title/Abstract]) OR (Premature Ventricular Complex[Title/Abstract]) OR (Ventricular Complex, Premature[Title/Abstract])
#3	“wenxin keli” [Supplementary Concept]
#4	“Metoprolol” [Mesh]
#5	(Toprol[Title/Abstract]) OR (Betaloc[Title/Abstract]) OR (Betaloc-Astra[Title/Abstract]) OR (Betaloc Astra[Title/Abstract]) OR (Betalok[Title/Abstract]) OR(Metoprolol Tartrate[Title/Abstract]) OR (Seloken[Title/Abstract]) OR (Spesicor[Title/Abstract]) OR (Spesikor[Title/Abstract]) OR (Metoprolol Succinate[Title/Abstract]) OR (Metoprolol CR-XL[Title/Abstract]) OR (Metoprolol CR XL[Title/Abstract]) OR (Toprol-XL[Title/Abstract]) OR (Toprol XL[Title/Abstract]) OR (Beloc-Duriles[Title/Abstract]) OR (Beloc Duriles[Title/Abstract]) OR (Lopressor[Title/Abstract])
#6	#4 OR #5
#7	“Randomized Controlled Trials as Topic” [Mesh]
#8	(Clinical Trials, Randomized[Title/Abstract]) OR (Trials, Randomized Clinical[Title/Abstract]) OR (Controlled Clinical Trials, Randomized[Title/Abstract])
#9	#7 OR #8
#10	#1 OR #2
#11	#10 AND #3 AND #6 AND #9

Retrieval strategy (all studies are searched based on the principles of PICOs).

### Inclusion criteria

#### Type of study

A randomized controlled trial of WXKL combined with metoprolol tartrate in the treatment of premature ventricular contractions. Randomized controlled trials without gender and language restrictions.

#### Research objects

The included patients with premature ventricular contractions need to be diagnosed by electrocardiogram or dynamic electrocardiogram, which is in line with the “Expert Consensus on Ventricular Arrhythmia” jointly issued by the European Heart Rhythm Association (EHRA), the American Heart Rhythm Society (HRS) and the Asia Pacific Heart Rhythm Society (APHRS) in August 2014 (4).

#### Interventions

The patients in the treatment group were given WXKL on the basis of the control group. The control group was treated with metoprolol tartrate tablets alone on the basis of conventional treatment.

Optimal medical therapy was defined as evidence-based stable doses of metoprolol tartrate tablets alone or in combination with stable doses of WXKL for at least 4 weeks.

#### Types of outcome measures

##### Primary outcomes

Effective clinical efficacy, Holter electrocardiogram improvement rate of premature ventricular contractions, and the incidence of adverse reactions.

#### Effective criteria

Refer to the “Guidelines for Clinical Research of Cardiovascular Drugs” formulated by the Ministry of Health's cardiovascular system drugs and clinical pharmacology base and the efficacy standards formulated by the 1979 Conference of Integrative Medicine (22–24).

##### The effective criteria of VPCs were as follows

Significant effect: 24-h dynamic electrocardiogram showed no premature beats or the number of premature beats decreased by more than 90% compared with before treatment. The subjective clinical symptoms were completely relieved, palpitations disappeared, chest tightness, chest pain, and other clinical symptoms disappeared or improved significantly.

Effective: A 24-h dynamic electrocardiogram showed that the number of premature beats was reduced by 50 to 90% compared with that before treatment. The subjective clinical symptoms were basically relieved, palpitations were significantly relieved, or other symptoms, such as chest tightness and chest pain, were partially relieved.

Invalid: The 24-h dynamic electrocardiogram showed that the number of premature beats decreased by <50% compared with that before treatment. The subjective clinical symptoms were not relieved, and symptoms such as chest tightness and chest pain were not significantly improved or even aggravated.


$$\begin{array}{l} The\;total\;effective\;rate\;=(the\;number\;of\;markedly\;effective\;cases\\ +the\;number\;of\;effective\;cases)/the\;total\;number\;of\;cases\;\times \;100\%. \end{array}$$

##### Safety indicators

Subjective discomfort caused by taking the drug includes bradycardia, atrioventricular block, hypotension, malignant vomiting, dizziness and fatigue, or liver and kidney function damage.

### Exclusion criteria

(1) The diagnostic criteria for ventricular premature beats are not specified, or other diagnoses are used as criteria. (2) There was only an abstract but no full text, and the main data were insufficiently reported. (3) Patients with ventricular premature beats caused by digitalis drug or other drug poisonings, water and electrolyte imbalance, and acid-base imbalance. (4) Patients with thyroid dysfunction; patients with severe liver, lung, kidney, and other organ dysfunction and hematological primary disease or psychosis; pregnant women; etc. (5) Patients with sick sinus syndrome and atrioventricular block above degree II; patients with systolic blood pressure <90 mm Hg (1 mm Hg = 0.133k Pa) or heart rate < 60 beats/min. (6) Patients with other heart diseases, such as rheumatic heart disease, cardiomyopathy, viral myocarditis, congenital heart disease, or pulmonary heart disease; patients with acute myocardial infarction, unstable angina pectoris, and cardiac function grade III, IV, or above and kinds of literature without cardiac function classification (patients complicated with coronary heart disease, but the kinds of literature with good cardiac function are not included).

### Data extraction and quality evaluation

#### Data extraction and management

EndNoteX9.1 software was used for literature management. Two investigators independently performed the literature search, study selection, and data extraction and screened and checked them according to the inclusion and exclusion criteria. If there were any objections, a third investigator was consulted to assist in resolution. Excel was used for data collation, including the basic information (title, first author, publication time), subject characteristics (age, course of disease, sample size, course of treatment), intervention methods, and outcome measures of the literature.

#### Subgroup analyses

Subgroup analyses were performed according to drug dose or the presence or absence of comorbidities.

#### Assessment of risk of bias in included studies

According to the Cochrane Reviewer's Handbook, six evaluation criteria of the quality of RCTs were used, which involved the generation of random sequence, randomization concealment, blinding method, the integrity of outcome data, selective reporting, and other biases.

Evidence was also evaluated based on the GRADE process to make recommendations for clinical practice. Three outcome indicators mainly consider the following five separate GRADE areas: risk of bias, inconsistency, indirectness, imprecision, and publication bias.

#### Measures of treatment effect

Data analysis was performed using Revman 5.4 software provided by the Cochrane Collaboration. Dichotomous data used risk ratios (RR) as the effect size, effect size values, and 95% confidence intervals (CI) as assessments. Statistical significance was considered when *P* < 0.05.

#### Assessment of heterogeneity

The heterogeneity of the included studies was tested using the χ2 test. If *I*^2^ 50% and *P* > 0.1, there was no statistical heterogeneity between studies, and a fixed-effects model was used for data analysis; if *I*^2^ > 50% and *P* < 0.1, there was statistical heterogeneity between studies, and randomization was used for data analysis. Effects models analyze the causes of heterogeneity. Subgroup analysis was used when there was clinical heterogeneity. Subgroups were divided according to sources of heterogeneity, such as intervention, drug dose, and comorbidities. If heterogeneity persisted, descriptive analysis was used.

#### Assessment of publication biases

If the number of included studies was >10 results, a funnel plot was used to analyze the potential for publication bias. When the funnel plot was symmetric, there was no reporting bias, and when it was asymmetrical, there was reporting bias.

#### Sensitivity analysis

Sensitivity analysis was performed using the “metainf” command of Stata 16 software.

## Results

### Description of included trials

#### Search process

Using the method of subject headings and free words, a total of 858 articles were searched from seven databases, and these articles were entered into EndNote in the form of a bibliography. After removing 435 duplicates, 423 possibly related articles were saved for reference and further evaluation. After evaluating the titles and abstracts, we excluded 388 articles. In the remaining 35 articles, we further excluded 24 articles after reviewing the full text. Finally, we included 11 articles for meta-analysis. The flowchart (Supplementary Figure 1) shows the search process and study selection.

#### Included studies

We included 11 studies in this review (25–35). The details are presented in Table 2. All studies were conducted in China and published in full. We did not find any unpublished studies.

**Table 2:** The details of the included studies.

**Serial number**	**Studies**	**Year**	**N (T/C)**	**Treatment group**	**Control group**	**Course (week)**	**Outcomes**
1	Chen and Shan (25)	2014	100 (50/50)	WXKL + Metalol tartrate	Metalol tartrate	4	a.b.c.d
2	Zhao (26)	2018	80 (40/40)	WXKL + Betaloc	Betaloc	4	a.b.c
3	Xu et al. (27)	2009	64 (32/32)	WXKL + Betaloc	Betaloc	4	a.b.c.d
4	Wang (28)	2017	88 (45/43)	WXKL + Betaloc	Betaloc	16	a.b.d
5	Xu (29)	2014	87 (43/44)	WXKL + Betaloc	Betaloc	4	a.b.c
6	Luo et al. (30)	2013	64 (32/32)	WXKL + Metalol tartrate	Metalol tartrate	4	a.b.c
7	Zhang et al. (31)	2009	103 (53/50)	WXKL + Betaloc	Betaloc	4	a.b.c
8	Wang et al. (32)	2014	108 (54/54)	WXKL + Betaloc	Betaloc	4	a.b.c.d
9	Wu (33)	2014	64 (32/32)	WXKL + Metoprolol	Metoprolol	4	a.b.c
10	Zhang (34)	2015	112 (54/58)	WXKL + Metoprolol	Metoprolol	4	a.b.c
11	Dai and Zhengdong (35)	2017	84 s(42/42)	WXKL + Metalol tartrate	Metalol tartrate	4	a.b.c

Outcomes: a. Clinical efficacy; b. 24 h electrocardiogram; c. Subjective adverse reactions (dizziness, nausea, etc.). d. Liver and kidney damage.

#### Participants

A total of 890 participants with VPCs were included in the 11 studies. The average sample size of the trials was 81 participants (ranging from 64 to 112 participants per trial). Gender was mentioned in 10 studies, including 405 women and 485 men. Among them, the study (29) only included women. In addition, the study (27) was divided into three groups, and the number of men and women in the two groups was not specified. Participants in six studies had coronary artery disease but had good heart function. The study (34) was on premature ventricular contractions after coronary stenting. The course of treatment in all 10 studies was 4 weeks, and in only one study (28), the period was 16 weeks. The age of the participants varied from 22 to 78 years old.

#### Interventions

All the 11 included study treatment groups used WXKL combined with metoprolol tablets on the basis of the control group, and the control group used metoprolol tablets alone on the basis of the basic treatment. Three studies (28, 29, 32) did not state the specific doses of WXKL, and one study (35) had different dosage units for WXKL.

#### Outcomes

All the 11 included studies reported the overall response rate of clinical efficacy and the overall effect of 24-h ECG improvement in premature ventricular contractions. Nine studies (25–35) reported adverse reactions, and the study (28) only mentioned liver and kidney function and did not describe other adverse reactions. The results are shown in Tables 3–5.

**Table 3:** Clinical efficacy of premature ventricular contractions.

**Studies**	**Groups (T/C)**	**The total effect (n)**	**Ineffective (n)**	**Total efficiency (%)**
Chen and Shan (25)	WXKL + Metalol tartrat	43	7	86
	Metalol tartrate	38	12	76
Zhao (26)	WXKL + Betaloc	38	2	95
	Betaloc	31	9	77.5
Xu et al. (27)	WXKL + Betaloc	29	3	90.6
	Betaloc	23	9	71.9
Wang (28)	WXKL + Betaloc	44	1	97.8
	Betaloc	32	11	74.4
Xu (29)	WXKL + Betaloc	42	1	97.67
	Betaloc e	36	8	81.82
Luo et al. (30)	WXKL + Metalol tartrate	29	3	90.6
	Metalol tartrate	18	14	56.2
Zhang et al. (31)	WXKL + Betaloc	51	2	96.23
	Betaloc	37	13	74
Wang et al. (32)	WXKL + Betaloc	47	7	90.6
	Betaloc	28	26	56.2
Wu (33)	WXKL + Metoprolol	30	2	93.7
	Metoprolol	20	10	66.7
Zhang (34)	WXKL + Metoprolol	49	5	90.7
	Metoprolol	43	15	74.1
Dai and Zhengdong (35)	WXKL + Metalol tartrate	38	4	90.5
	Metalol tartrate	28	14	66.7

**Table 4:** The frequency improvement effect of VPCs within 24 h.

**Studies**	**Groups (T/C)**	**The total effect (n)**	**Ineffective (n)**	**Total efficiency (%)**
Chen and Shan (25)	WXKL + Metalol tartrat	42	8	84
	Metalol tartrate	38	12	76
Zhao (26)	WXKL + Betaloc	37	3	92.5
	Betaloc	29	11	27.5
Xu et al. (27)	WXKL + Betaloc	30	2	93.8
	Betaloc	18	14	56.3
Wang (28)	WXKL + Betaloc	43	2	95.6
	Betaloc	31	12	72.1
Xu (29)	WXKL + Betaloc	41	2	95.35
	Betaloc e	35	9	79.55
Luo et al. (30)	WXKL + Metalol tartrate	28	4	87.5
	Metalol tartrate	20	12	62.5
Zhang et al. (31)	WXKL + Betaloc	50	3	93.34
	Betaloc	39	11	78
Wang et al. (32)	WXKL + Betaloc	45	9	83.3
	Betaloc	31	19	57.4
Wu (33)	WXKL + Metoprolol	29	3	90.6
	Metoprolol	18	12	60
Zhang (34)	WXKL + Metoprolol	48	6	88.9
	Metoprolol	40	18	74.1
Dai and Zhengdong (35)	WXKL + Metalol tartrate	36	6	85.7
	Metalol tartrate	27	15	64.3

**Table 5:** The incidence of adverse reactions.

**Studies**	**Groups (T/C)**	**Total occurrences (** ***n*** **)**	**Total incidence (%)**
Chen and Shan (25)	WXKL + Metalol tartrat	0	0
	Metalol tartrate	0	0
Zhao (26)	WXKL + Betaloc	4	10
	Betaloc	13	32.5
Xu et al. (27)	WXKL + Betaloc	0	0
	Betaloc	3	9.4
Wang (28)	WXKL + Betaloc	0	0
	Betaloc e	0	0
Xu (29)	WXKL + Metalol tartrate	0	0
	Metalol tartrate	2	6.3
Luo et al. (30)	WXKL + Betaloc	2	3.8
	Betaloc	3	6
Zhang et al. (31)	WXKL + Betaloc	2	3.7
	Betaloc	2	3.7
Wang et al. (32)	WXKL + Metoprolol	4	12.5
	Metoprolol	2	6.7
Wu (33)	WXKL + Metoprolol	3	5.6
	Metoprolol	3	5.2
Zhang (34)	WXKL + Metalol tartrate	3	7.1
	Metalol tartrate	4	9.5

#### Risk of bias assessment

All trials provided limited information on trial design and methods. There are 4 trials that clearly describe the method of generating random sequences, and the remaining 9 trials only mention randomness without describing the method of generating random sequences in detail. In addition, allocation concealment and blinding (participants, personnel, outcome assessment) were not mentioned in any of the 11 included studies, although the included studies did not restrict allocation concealment and blinding. In the (28) study, the reports of adverse reactions only mentioned liver and kidney function, and the rest of the adverse reactions were not clearly stated, and there was a possibility of selection bias, as shown in Supplementary Figures 2–11.

### Effects of Interventions

#### Effective clinical efficacy

All 11 included studies had detailed clinical efficacy rates. The heterogeneity test showed that the homogeneity among the studies was good (*P* = 0.79 > 0.1, *I*^2^=8 <50%). The fixed-effect model was used to combine the effect size. The results showed that the clinical efficacy of the treatment group was higher than that of the treatment group. In the control group, the difference was statistically significant (RR = 1.32, 95% CI: [1.24, 1.40], *P* < 0.00001), as shown in Supplementary Figure 12.

#### Twenty-four-hour ECG improvement rate of PVCs

All 11 included studies detailed improvements in the rate of premature ventricular contractions on ECG. The heterogeneity test showed that the homogeneity among the studies was good (*P* = 0.46>0.1, *I*^2^ = 0 <50%), and a fixed-effect model was used to combine the effects. The results showed that the effective rate of improving the rate of premature ventricular beats displayed on the ECG in the treatment group was higher than that in the control group, and the difference was statistically significant (RR = 1.32, 95% CI: [1.23, 1.41], *P* < 0.00001; Supplementary Figure 13).

#### The incidence of adverse reactions

The 11 included studies all involved the occurrence of adverse reactions, but (28) of them only briefly explained the status of liver and kidney function and did not clearly explain the subjective discomfort of participants (bradycardia, atrioventricular block, low blood pressure, malignant vomiting, dizziness, fatigue, etc.) occurrence of other adverse reactions. Therefore, only 10 studies were included in the assessment of adverse reaction rates. The heterogeneity test showed that the homogeneity between the studies was good (*P* = 0.53>0.1, *I*^2^ = 0 <50%). The fixed-effect model was used to combine the effect size, and the number of adverse reactions in the treatment group (*n* = 18) was higher than that in the control group (*n* = 32). Only the (33) study of the included studies had two more adverse reactions in the treatment group than in the control group. Meta-analysis results showed (RR = 0.61, 95% CI: [0.35,1.05], P = 0.08) that there was no difference in the incidence of adverse reactions between the two groups. However, various studies have pointed out that after reducing the dose of metoprolol, mild adverse reactions (such as bradycardia, nausea, dizziness, etc.) in the two groups were relieved spontaneously, but the more serious adverse reactions (such as acute myocardial infarction, cardiac failure, etc.) less than the control group. Therefore, WXKL can reduce the occurrence of adverse reactions (Supplementary Figure 14).

#### Subgroup analysis of clinical efficacy

All 11 included studies reported clinical response rates. There were 440 effective cases in the treatment group and 334 effective cases in the control group. All included studies reported the presence or absence of coexisting coronary heart disease. The 11 included studies were pooled using a fixed-effect model. The results showed that there was no statistical heterogeneity among the studies (*P* = 0.37, *I*^2^ = 8%), and the total clinical response rate in the treatment group was higher than that in the control group (RR = 1.32, 95% CI:[1.24, 1.40], *P* < 0.0001). There was no heterogeneity between the two subgroups (*P* = 0.71, *I*^2^ = 0%). Combined studies without coronary heart disease showed no significant statistical heterogeneity among the studies (*P* = 0.29, *I*^2^ = 19%). Combined analysis using a fixed effect model showed that the clinical effective rate in the treatment group was higher than that in the control group (RR = 1.30, 95% CI: [1.19,1.43], *P* < 0.00001). There was no significant statistical heterogeneity among the studies (*P* = 0.34, *I*^2^ = 11%), and a fixed effect model was used for analysis. The results showed that the total clinical effective rate in the treatment group was higher than that in the control group (RR = 1.33, 95% CI: [1.2, 1.46], *P* < 0.00001). The result is shown in Supplementary Figure 15.

Four of the included studies (28, 29, 32, 35) did not clearly describe the dosage of WXKL. Therefore, the remaining seven studies were combined and analyzed using a fixed effect model. There was no statistical heterogeneity between studies (*P* = 0.51, *I*^2^ = 0%). The total clinical response rate in the treatment group was higher than that in the control group (RR = 1.27, 95% CI: [1.17, 1.37], *P* < 0.00001). Due to the significant heterogeneity between the two subcomponents with WXKL doses of 15 g/d and 27 g/d, the source of heterogeneity may be caused by the large difference in sample sizes between the two groups, so the combined analysis was not conducted. The results of both groups indicated that the clinical effective rate of the WXKL treatment group was higher than that of the control group (*P* < 0.05). The result is shown in Supplementary Figure 16.

### Publication biases

Bias funnel plot analysis was performed on the meta-analysis results of clinical efficacy, as shown in Supplementary Figure 17. The funnel plot was asymmetrical, suggesting the possibility of selection bias. That is, partially negative results were unpublished or not published. The 11 included studies all mentioned that after reducing the dose of metoprolol, the adverse reactions were reduced or even alleviated, but it was not clear whether the clinical efficacy of these participants was included in the final efficacy study. There may be selection bias.

### Sensitivity analysis and regression analysis

Sensitivity analysis of results from clinical efficacy studies showed a minimum of 1 for all studies, indicating that there was no significant difference in results when any studies were excluded. It was proven that the total effective rate of clinical efficacy was low sensitivity, with good stability and reliability, and the analysis results were stable and reliable (see Table 6 and Supplementary Figure 18).

**Table 6:** Sensitivity analysis results of clinical efficacy.

**Study omitted**	**Estimate**	**[95% Conf. Interval]**	
Chen and Shan (25)	1.3001033	1.2194467	1.3860947
Wu (33)	1.2724231	1.1956913	1.3540791
Xu et al. (27)	1.2879473	1.2043622	1.3773334
Dai and Zhengdong (35)	1.2802169	1.1979548	1.3681281
Xu (29)	1.3011975	1.2171762	1.3910189
Luo et al. (30)	1.2715725	1.1953256	1.3526829
Zhang et al. (31)	1.2844718	1.1982825	1.3768604
Wang et al. (32)	1.2647605	1.188412	1.346014
Zhang (34)	1.2939441	1.2086096	1.3853036
Zhao (26)	1.2931718	1.2080935	1.3842417
Wang (28)	1.2826974	1.1973552	1.3741225
Combined	1.2824938	1.2064571	1.3633228

Subgroups with or without coronary heart disease were sensitized using a one-by-one exclusion method. After excluding the two studies (30, 32), the *I*^2^ values of both the group without coronary heart disease and the group with coronary heart disease decreased slightly (*I*^2^ = 0%).

The two subgroups with different doses of WXKL were not combined due to significant heterogeneity. Sensitivity analysis was performed using a one-by-one elimination method. Excluding this study (25) the intergroup results were (*P* = 0.40, *I*^2^ = 0%). In this study (25), it was clearly stated that the long course of disease in the included cases may be the cause of heterogeneity.

Regression analysis of different doses of WXKL, coronary heart disease and sex showed *P* > 0.05, so there was no significant effect on the total clinical response rate.

### GRADE quality evaluation

#### Total effective rate of clinical efficacy

All 11 included studies (*n* = 954) that used WXKL in combination with metoprolol, compared with metoprolol, 10 of which had a 4-week treatment course and 1 of which had a 16-week treatment course (28). To observe the disease response rate defined by clinical symptom relief and reduced frequency and frequency of premature ventricular beats, the results suggested that WXKL combined with metoprolol had a higher response rate compared with the control group, and the difference between groups was statistically significant (RR = 1.32, 95% CI [1.24, 1.4], *P* < 0.00001). The evidence is shown in Supplementary Figure 19.

#### Improvement rate of the ventricular premature beat of dynamic electrocardiogram

All 11 included studies *(n* = 954) used WXKL in combination with metoprolol vs. metoprolol for 4 weeks, and one study (28) had a treatment course of 16 weeks. Observation of the disease improvement rate based on the definition of reduced frequency and number of premature ventricular beats showed that WXKL combined with metoprolol was more effective than the control group, and the difference between groups was statistically significant (RR = 1.32, 95% CI: [1.23, 1.41], *P* < 0.00001). The evidence is shown in Supplementary Figure 20.

#### Incidence of adverse reactions

All nine included studies (25–27, 29–35) (*n* = 866) used WXKL in combination with metoprolol compared with metoprolol for 4 weeks. To observe the frequency of impairment of liver and kidney function based on conscious symptoms. There were two studies (25), and the frequency of occurrence between the experimental group and control group was 0 (29), so a total of seven studies were included for analysis. The results showed that WXKL combined with metoprolol (*n* = 18) showed no statistically significant difference compared with the control group (*n* = 30) (RR = 0.61, 95% CI [0.35, 1.05], *P* = 0.08). However, according to the severity of adverse reactions described in each study, the experimental group had higher safety than the control group. The evidence is shown in Supplementary Figure 21.

## Discussion

### The effectiveness of WXKL

Premature ventricular contractions are the most common arrhythmia, mostly due to increased pacemaker automaticity, circular reentry, or focal microreentry, triggering activity (36). There are many clinical causes of arrhythmia, which are not only related to heart disease but also related to human neurohumoral factors (37). Premature ventricular contractions can be divided into functional and organic contractions. Most people with functional premature beats are asymptomatic, and they are given health education and reassurance without the need for drug treatment. However, some people's attacks can affect the quality of life of patients and even endanger their lives. At this time, drug treatment is needed. Most of the antiarrhythmic drugs commonly used in clinics have proarrhythmic side effects and are prone to drug tolerance. Therefore, there is an urgent need to seek a drug with fewer side effects in clinical practice. WXKL is a traditional Chinese medicine mechanism with an antiarrhythmic effect. WXKL is a pure Chinese herbal mechanism with antiarrhythmic effects that has the effects of class I, III, and IV antiarrhythmic drugs and regulates a variety of ion channels. Studies have shown that WXKL can safely and effectively treat arrhythmias by selectively inhibiting the steady-state inactivation of atrial sodium ion channels, thereby reducing the resting membrane potential of cells (38). It can also selectively inhibit the fast sodium current and generate a refractory period after atrial repolarization, clearly and effectively terminate and prevent atrial fibrillation, improve arrhythmia, and selectively inhibit the late sodium current on Purkinje cells to play a role in the treatment of arrhythmia (39). Moreover, WXKL can regulate Ito by antagonizing the effect of angiotensin II (Ang II) on the potassium current of the atrial myocyte membrane (40). In addition, WXKL has the effect of resisting myocardial ischemia–reperfusion injury. Clinical studies have found that combined use of WXKL based on conventional guideline treatment can improve myocardial ischemia, downregulate plasma BNP levels, and increase HRV, thereby improving clinical prognosis in recent times (41).

WXKL is composed of Codonopsis Radix, Polygonati Rhizoma, Notoginseng Radix Et Rhizoma, Ambrum, and Nardostachyos Radix Et Rhizoma. The combination of various drugs has the effect of nourishing qi, nourishing yin, promoting blood circulation, and removing blood stasis. Modern pharmacological studies have shown that Codonopsis can enhance cardiac output, resist platelet aggregation, protect myocardial cells, and improve myocardial energy metabolism (42). Codonopsis Radix has antioxidant functions, inhibiting calcium overload and regulating vascular endothelial cells to prevent reperfusion injury (43). The succinic acid contained in Ambrum can enhance the body's immunity and maintain stable acidity. Polygonati Rhizoma can increase coronary blood volume and reduce lipids. Notoginseng Radix Et Rhizoma pine extract has a concentration-dependent blocking effect on the currents of Na, Ito, LCA-L, etc., can prolong the action potential, and can effectively inhibit the reentrant excitation (42). Studies have found that the Notoginseng Radix Et Rhizoma extract in WXKL can act on the cell signal transduction pathways of cyclic adenosine monophosphate (cAMP) and protein kinase A (PKA), inhibit calcium ion channels, and cause tachyarrhythmias. Symptoms improved in rats (44).

The treatment of ventricular premature beats with WXKL combined with metoprolol tartrate can give full play to the synergistic effect of drugs, enhance the antiarrhythmic effect, and reduce the dosage of metoprolol to reduce the occurrence of adverse reactions. Patients can also adhere to long-term medication to achieve the effect of improving symptoms and signs. The results of this study showed the total clinical efficacy (RR = 1.32, 95% CI: [1.24, 1.40], *P* < 0.00001) and the improvement effect of premature ventricular beats (RR = 1.32, 95% CI: [1.23, 1.41], *P* < 0.00001), suggesting that WXKL combined with metoprolol tartrate has good efficacy in the treatment of premature ventricular beats. Although the incidence of adverse reactions (RR = 0.61, 95% CI: [0.35, 1.05], *P* = 0.08), all the included studies clearly indicated that after reducing the metoprolol dose, mild adverse reactions (such as bradycardia, nausea, dizziness, etc.) in the two groups were spontaneously relieved, but severe adverse reactions (such as acute myocardial infarction, heart failure, etc.) in the treatment group were less than those in the control group. Therefore, WXKL causes fewer adverse reactions than metoprolol and can reduce the adverse reactions caused by metoprolol. Therefore, WXKL has good security. Therefore, WXKL is worthy of clinical promotion and further development and application.

### The safety of WXKL

At present, for the treatment of premature ventricular contractions, radiofrequency ablation has been widely used in clinical practice, but some researchers have indicated that radiofrequency ablation is more effective than drugs in the treatment of frequent premature ventricular contractions with obvious symptoms or cardiac insufficiency (45, 46). However, there may be hidden damage to the structure and function of the heart in the early stage of premature ventricular contraction (47), which requires early identification and appropriate drug treatment and consideration of adverse drug reactions and safety of drug application. All studies included in this study involved adverse drug reactions, involving a total of 866 patients. There were 18 cases of adverse reactions in the treatment group and 32 cases of adverse reactions in the control group. Among the adverse reactions, mild symptoms such as dizziness, nausea, and bradycardia occurred in both groups. The adverse reactions of most patients in the treatment group could be relieved spontaneously, and the other patients were the same as those in the control group, which could be relieved after reducing the dose of metoprolol. In the patients with coronary heart disease, severe heart failure, myocardial infarction, and other serious diseases occurred, and the treatment group had fewer severe cases than the control group, and the degree was milder than that of the control group. Therefore, no adverse reactions to WXKL-increasing drugs were observed, and the adverse reactions caused by metoprolol tablets were alleviated to a certain extent, with good safety.

The electrophysiology of myocardial cells is influenced by multiple current channels. If these current movement occurs disorder, cardiac arrhythmia would come into being (48). Currently, most western antiarrhythmic drugs are single current channel blockers which is the cause of many adverse reactions. Considering various factors, metoprolol tartrate has more advantages than other antiarrhythmic drugs, so we chose metoprolol tartrate as the control group. WXKL is a kind of traditional Chinese medicine which has beneficial effects on modulating variety of ion channels against cardiac arrhythmia (38). WXKL could regulate several current channels just right, which may be the main reason to reduce the occurrence of adverse reactions and have better safety.

### Dose-effect relationship

Dose analysis in subgroup analysis showed that WXKL may be dose-dependent in treatment. However, since there are only two studies with low doses, which are less than those with middle doses, further studies need to be expanded for further verification.

### Practicability and clinical significance of this study

Analyses of the included studies suggest that WXKL may be effective and safe for the treatment of premature ventricular contractions. Compared with other antiarrhythmic Western medicines, WXKL is more preferential and cost-effective, with stable synergistic effects and few side effects. In addition, through this systematic review, we found some shortcomings, which should be noted in future clinical trials. First, an appropriate random method was chosen, and split items were hidden to ensure comparability and reduce selection bias. Double-blind or triple-blind should be used. Second, the duration of drug intervention should be extended to observe the effects of long-term medication on the cardiac structure. Third, the observation indicators should also include cardiac ejection fraction, heart failure index, BNP, etc., as well as extend the observation range of adverse reactions. Fourth, clinical trials should be conducted overseas to observe individual differences between regions.

## Conclusion

The efficacy and safety of WXKL combined with metoprolol tartrate in the treatment of VPC are better than those of metoprolol tartrate alone. However, the included studies also had limitations and deficiencies in trial design (no blinding method, single country, limited scope of adverse reaction observation, etc.) and may have publication bias, resulting in relatively insufficient high-quality evidence. Further larger-scale, multicenter/country, longer follow-up, and higher-quality RCTs are needed to more fully verify the efficacy and safety of WXKL combined with metoprolol tartrate in the treatment of premature ventricular contractions.

## Data availability statement

The original contributions presented in the study are included in the article/Supplementary material, further inquiries can be directed to the corresponding author/s.

## Author contributions

Conception and design: PH, YL, and PM. Data collection, data analysis, and manuscript drafting: PH. Data extraction: PH and YL. Data validation: JC, JX, YS, and GC. Email trial registrant manuscript verification: PM. All authors contributed to the article and approved the submitted version.

## Funding

The present work was supported by grants from the National Natural Science Foundation of China (No. 81173365).

## Conflict of interest

The authors declare that the research was conducted in the absence of any commercial or financial relationships that could be construed as a potential conflict of interest.

## Publisher's note

All claims expressed in this article are solely those of the authors and do not necessarily represent those of their affiliated organizations, or those of the publisher, the editors and the reviewers. Any product that may be evaluated in this article, or claim that may be made by its manufacturer, is not guaranteed or endorsed by the publisher.

## Abbreviations

WXKL: Wenxin Keli
PVC/PVCs: premature ventricular contraction
ECG: electrocardiogram
HF: heart failure
EHRA: European Heart Rhythm Association
HRS: Heart Rhythm Society
APHRS: Asia Pacific Heart Rhythm Society
RCTs: randomized controlled trials
RR: risk ratio
CVDs: cardiovascular diseases
CNKI: China National Knowledge Infrastructure
VIP: Chinese Scientific Journal Database
BNP: brain natriuretic peptide
Ang II: angiotensin II
HRV: heart rate variability
cAMP: cyclic adenosine monophosphate
PKA: protein kinase A.
