# Supplementary material for: Efficacy and safety of Wenxin Keli combined with metoprolol tartrate in the treatment of premature ventricular contractions: A systematic review and meta-analysis
Source: Front Cardiovasc Med. 2022 Jul 29;9:952657. doi: 10.3389/fcvm.2022.952657 (PMC9372502; doi:10.3389/fcvm.2022.952657)
Supplement: Supplementary file 1 [file Data_Sheet_1.docx]

| **Section and Topic** | **Item #** | **Checklist item** | **Location where item is reported** |
| --- | --- | --- | --- |
| **TITLE** | | |  |
| Title | 1 | The report is identified as a systematic review and meta-ananlysis. |  |
| **ABSTRACT** | | |  |
| Abstract | 2 | The abstract includes Objiective, methods, results, and conclusions. |  |
| **INTRODUCTION** | | |  |
| Rationale | 3 | Describe in the Introduction. |  |
| Objectives | 4 | Stated in the Introduction. |  |
| **METHODS** | | |  |
| Eligibility criteria | 5 | The inclusion and exclusion criteria for this study are described in detail in lines 249-371. of the Methods section. |  |
| Information sources | 6 | The retrieval method and the 7 databases retrieved are described in detail in lines 232-246. |  |
| Search strategy | 7 | The search strategy is presented in Table 1. |  |
| Selection process | 8 | The selection of studies for inclusion and the work of the participants are described in lines 401-466. |  |
| Data collection process | 9 | The selection of studies for inclusion and the work of the participants are described in lines 404-413. |  |
| Data items | 10a | The selection of studies for inclusion and the work of the participants are described in lines 401-466. |  |
|  | 10b | The interventions, research subjects, etc. included in the study are described in lines 249-466, and funding sources are described after the conclusion of the article. |  |
| Study risk of bias assessment | 11 | Lines416-466 describe how the researchers used the tools to perform various assessments of the included studies and to address biases. |  |
| Effect measures | 12 | In lines 435-440 describes the data types, effect sizes, and effect measures of the included studies. |  |
| Synthesis methods | 13a | In lines 472-489 describe the search process for this study and the basic characteristics of the included studies. (Figure.1,Table 1) |  |
|  | 13b | In lines 472-489 describe the search process for this study and the basic characteristics of the included studies. |  |
|  | 13c | The methods and outcome analyses of the outcome measure meta-analysis are described in lines 468-911 and shown separately in figures. |  |
|  | 13d | Methods and outcome analyses of the meta-analysis of outcome measures are described in line 468-911. |  |
|  | 13e | Statistical analysis of the 3 outcomes of this study showed no significant heterogeneity. A subgroup analysis was performed for the overall response rate. In the subgroup analysis, a group of results showed heterogeneity, and sensitivity analysis and regression analysis were carried out. And a GRADE score was performed in lines 867-880 of this study to assess the effect across studies. |  |
|  | 13f | In lines 825-848 of this study, sensitivity analyses and regression analyses were performed on the clinical efficacy rates to assess the robustness of the results. |  |
| Reporting bias assessment | 14 | Bias analysis of included studies is described in lines 810-821. Figure 17 |  |
| Certainty assessment | 15 | The methodology used is described in lines 628-807 describing each outcome measure. |  |
| **RESULTS** | | |  |
| Study selection | 16a | The procedure for screening studies is described in lines 472-481.Figure.1. |  |
|  | 16b | The process and reasons for including and excluding literature are explained in lines 472-488. |  |
| Study characteristics | 17 | Lines 491-522 assess study participants, interventions, and treatment outcomes.Table 3,Table 4,Table 5 |  |
| Risk of bias in studies | 18 | Risk of bias assessment for each study is described in lines 526-583. Figure 2, Figure 3-Figure11. In lines 867-880 of this study, grade evaluation was carried out on each outcome index,and The summary of the outcome is shown in the figure 19, Figure 20, Figure 21. |  |
| Results of individual studies | 19 | Statistical analysis of outcome measures in lines 629-807, Figure 12, Figure 13, Figure 14, Figure15, Figure 16. |  |
| Results of syntheses | 20a | Bias analysis of included studies is described in lines 811-821. Figure 17. |  |
|  | 20b | In the lines 629-911, a meta-analysis of each outcome measure was carried out and a detailed description was given. |  |
|  | 20c | In the lines 629-911 shows that there is no significant heterogeneity in each outcome index. Subgroup analysis is carried out on the main outcome index, and one subgroup analysis is heterogeneous. Sensitivity analysis and regression analysis(lines 825-828) are carried out on it to evaluate the impact of the differences included in the study on the overall index. |  |
|  | 20d | In the meta-analysis in lines 825-848, a sensitivity analysis and regression analysis was performed to assess the robustness of the results. |  |
| Reporting biases | 21 | Part publication biases (lines 810-822)performed a bias analysis. |  |
| Certainty of evidence | 22 | The results of each assessment are analyzed in section (Effects of Interventions), and a sensitivity analysis and regression analysis are performed in section Sensitivity analysis and regression analysis. |  |
| **DISCUSSION** | | |  |
| Discussion | 23a | The sections (The effectiveness of WXKL, The safety of WXKL, and Dose-effect relationship) describe the research basis of this study and the comparative analysis of the research results. |  |
|  | 23b | Section discussion describes the evidence for the study and the limitations of each section. |  |
|  | 23c | Weaknesses and limitations of the included studies are described in detail in section Practicability and clinical significance of this study. . |  |
|  | 23d | Practicability and clinical significance of this study of this study are described in section Practicability and clinical significance of this study. |  |
| **OTHER INFORMATION** | | |  |
| Registration and protocol | 24a | Registered name: Efficacy and safety of Wenxin keli combined with metoprolol tartrate in the treatment of premature ventricular contractions: a systematic review and meta-analysis.  The protocol for this study has been registered in the International Systematic Prospective Register (PROSPERO, 2022 CRD42022329403). |  |
|  | 24b | PROSPERO |  |
|  | 24c | Consistent with the plan at the time of registration. |  |
| Support | 25 | The present work was supported by grants from the National Natural Science Foundation of China. |  |
| Competing interests | 26 | This article has no conflict of interest. |  |
| Availability of data, code and other materials | 27 | The figures and tables presented in the text extract and analyze the data of the included studies, and the relevant retrieval strategies are shown in Table1-5. These are publicly available. |  |

*From:*  Page MJ, McKenzie JE, Bossuyt PM, Boutron I, Hoffmann TC, Mulrow CD, et al. The PRISMA 2020 statement: an updated guideline for reporting systematic reviews. BMJ 2021;372:n71. doi: 10.1136/bmj.n71

For more information, visit: <http://www.prisma-statement.org/>
