# Supplementary figures and images for: Efficacy and safety of Wenxin Keli combined with metoprolol tartrate in the treatment of premature ventricular contractions: A systematic review and meta-analysis
Source: Front Cardiovasc Med. 2022 Jul 29;9:952657. doi: 10.3389/fcvm.2022.952657 (PMC9372502; doi:10.3389/fcvm.2022.952657)

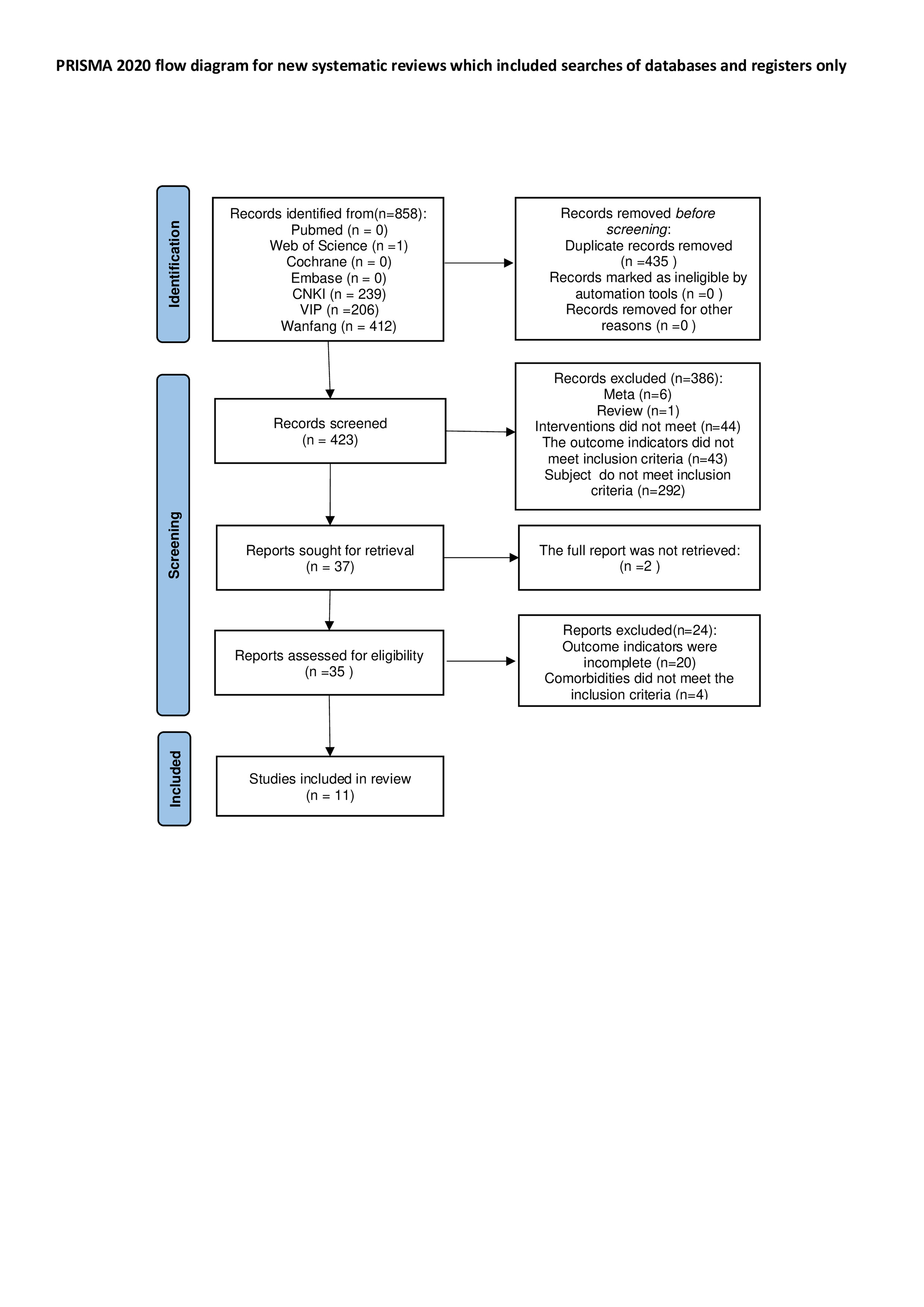

Supplement: Supplementary file 2 [file Data_Sheet_1.zip › Figures/Figure 1. Process of searching and screening studies..tif]

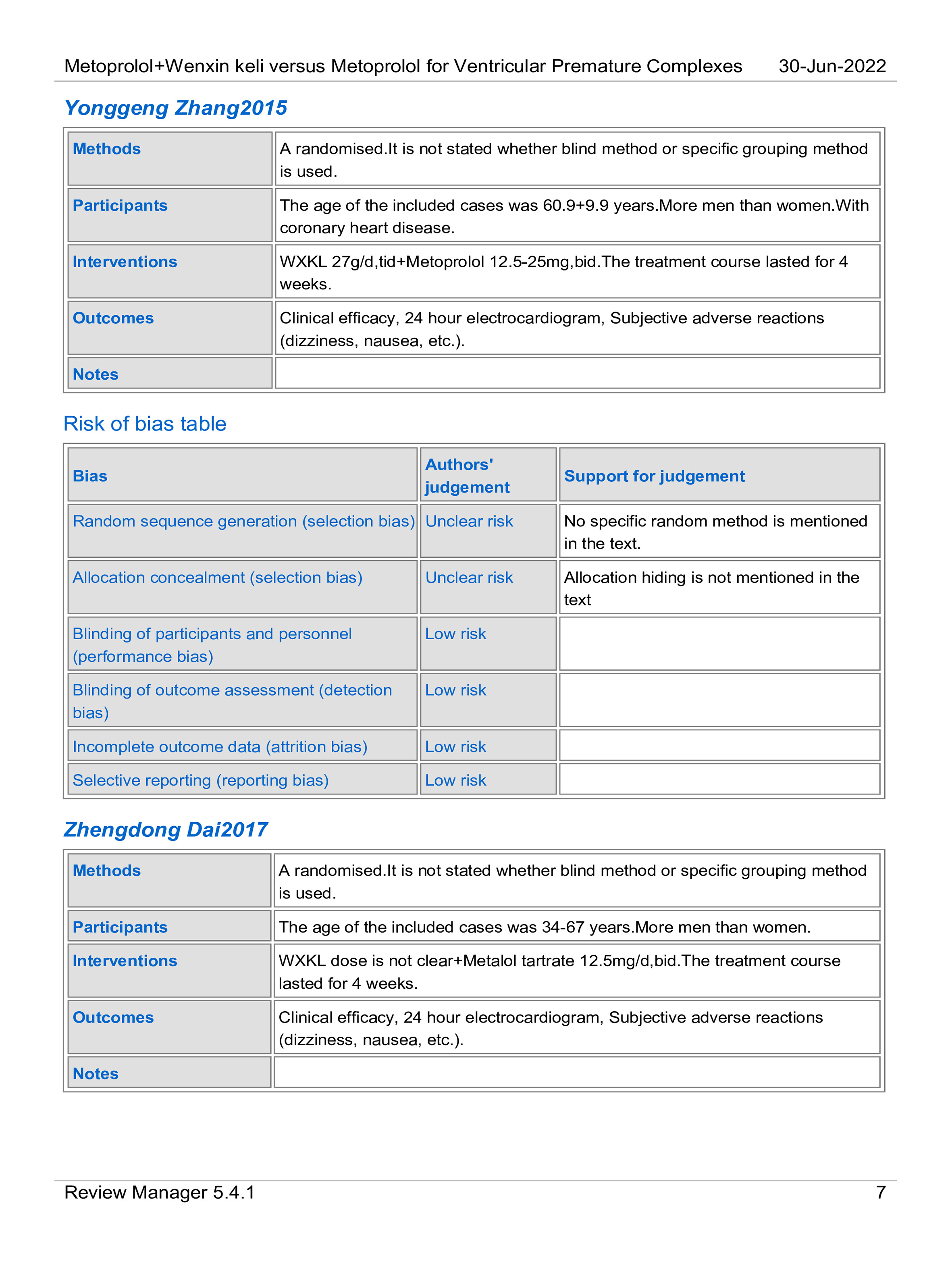

Supplement: Supplementary file 2 [file Data_Sheet_1.zip › Figures/Figure 10 Risk of bias table .tif]

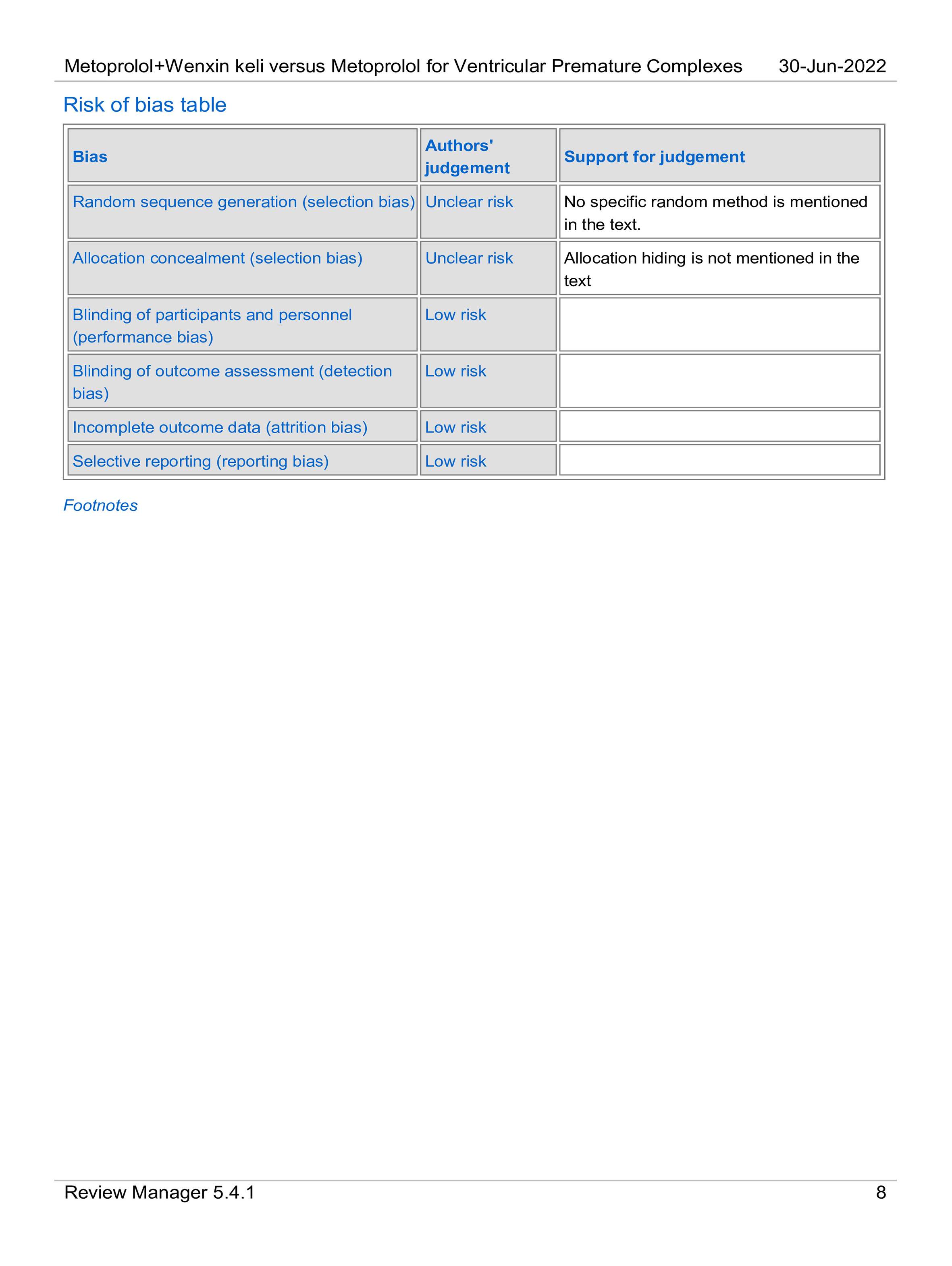

Supplement: Supplementary file 2 [file Data_Sheet_1.zip › Figures/Figure 11 Risk of bias table .tif]

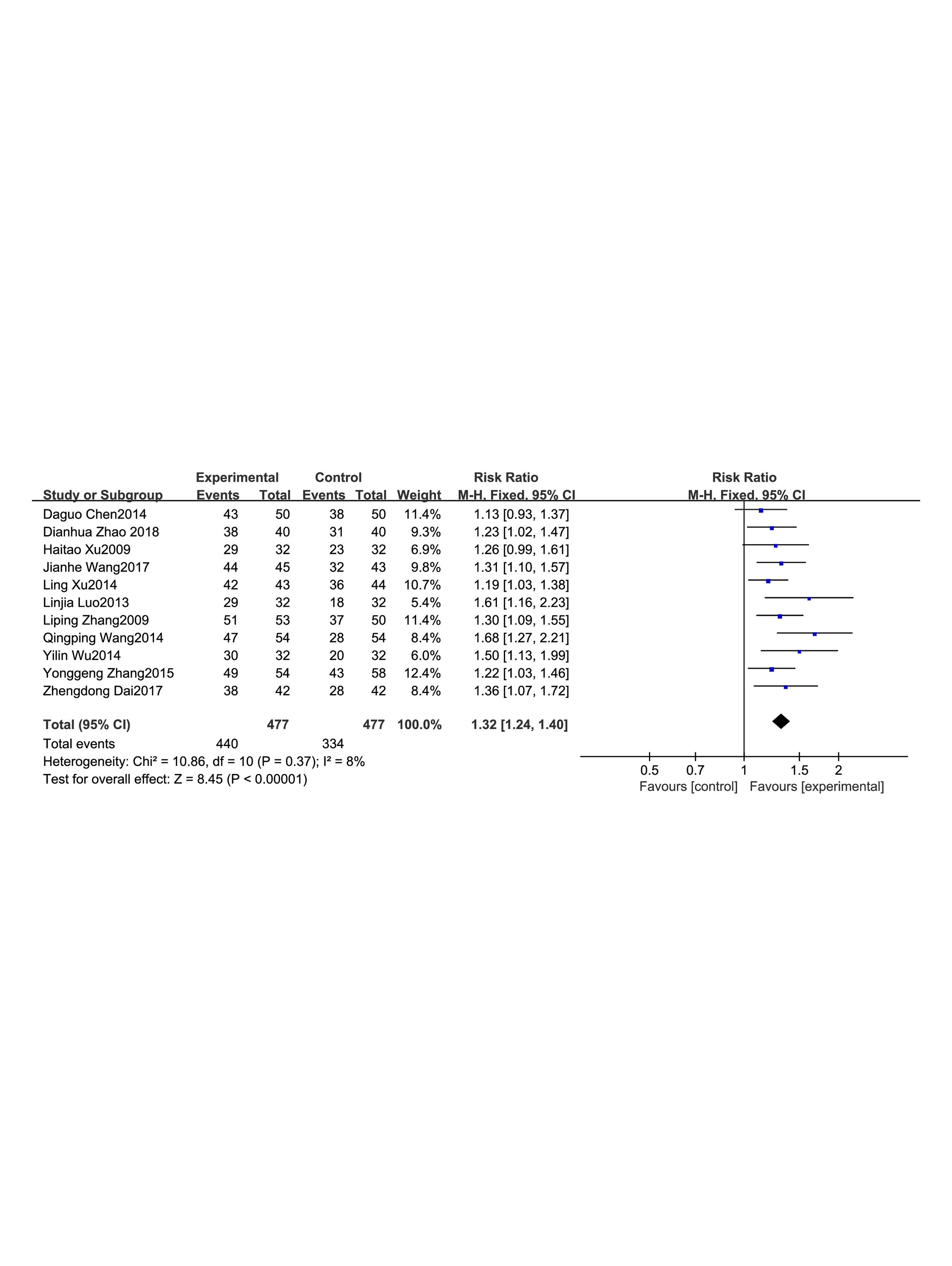

Supplement: Supplementary file 2 [file Data_Sheet_1.zip › Figures/Figure 12 The forest plot of effective clinical efficacy.tif]

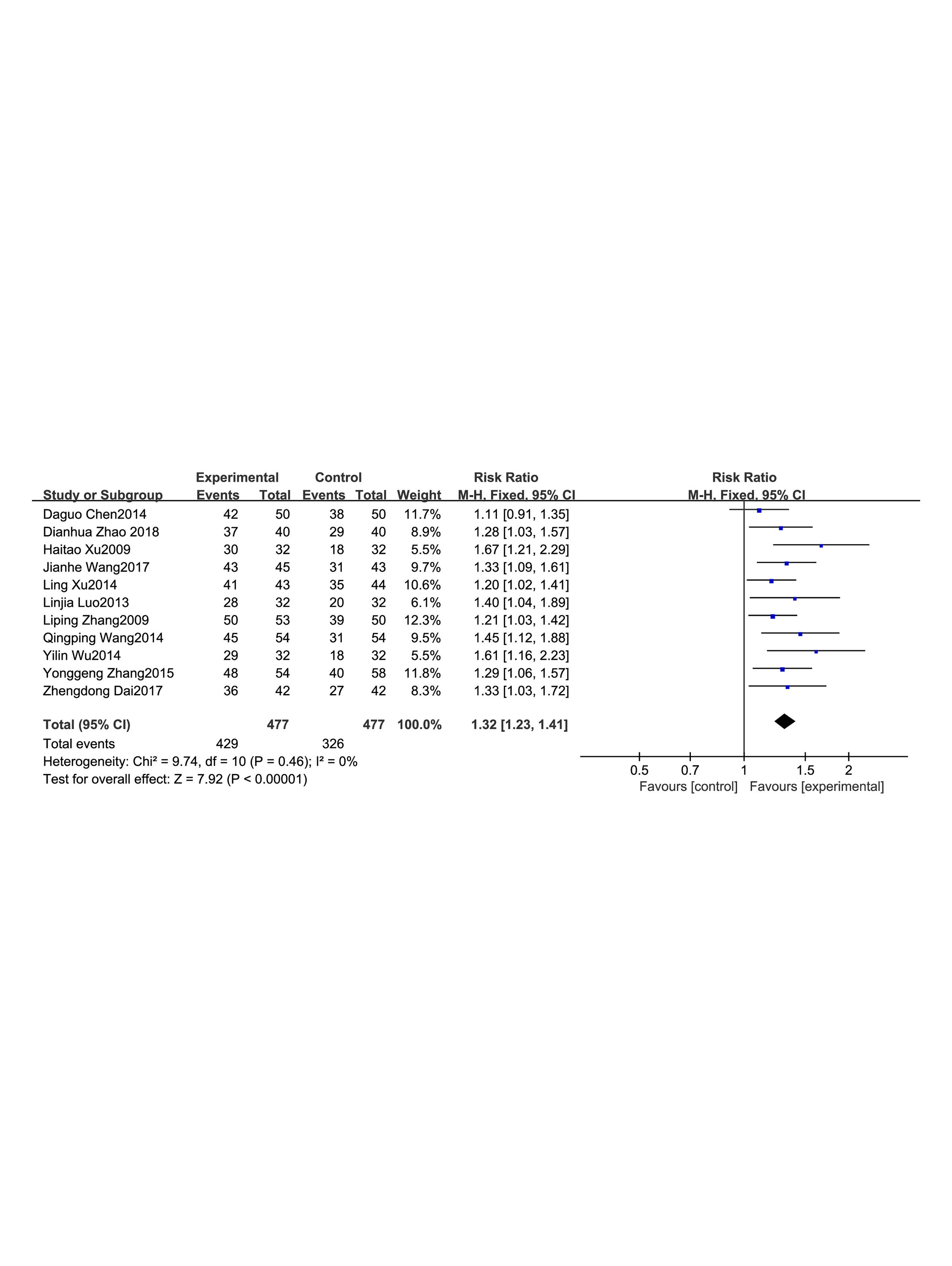

Supplement: Supplementary file 2 [file Data_Sheet_1.zip › Figures/Figure 13 The forest plot of 24-hour ECG improvement rate of PVCs.tif]

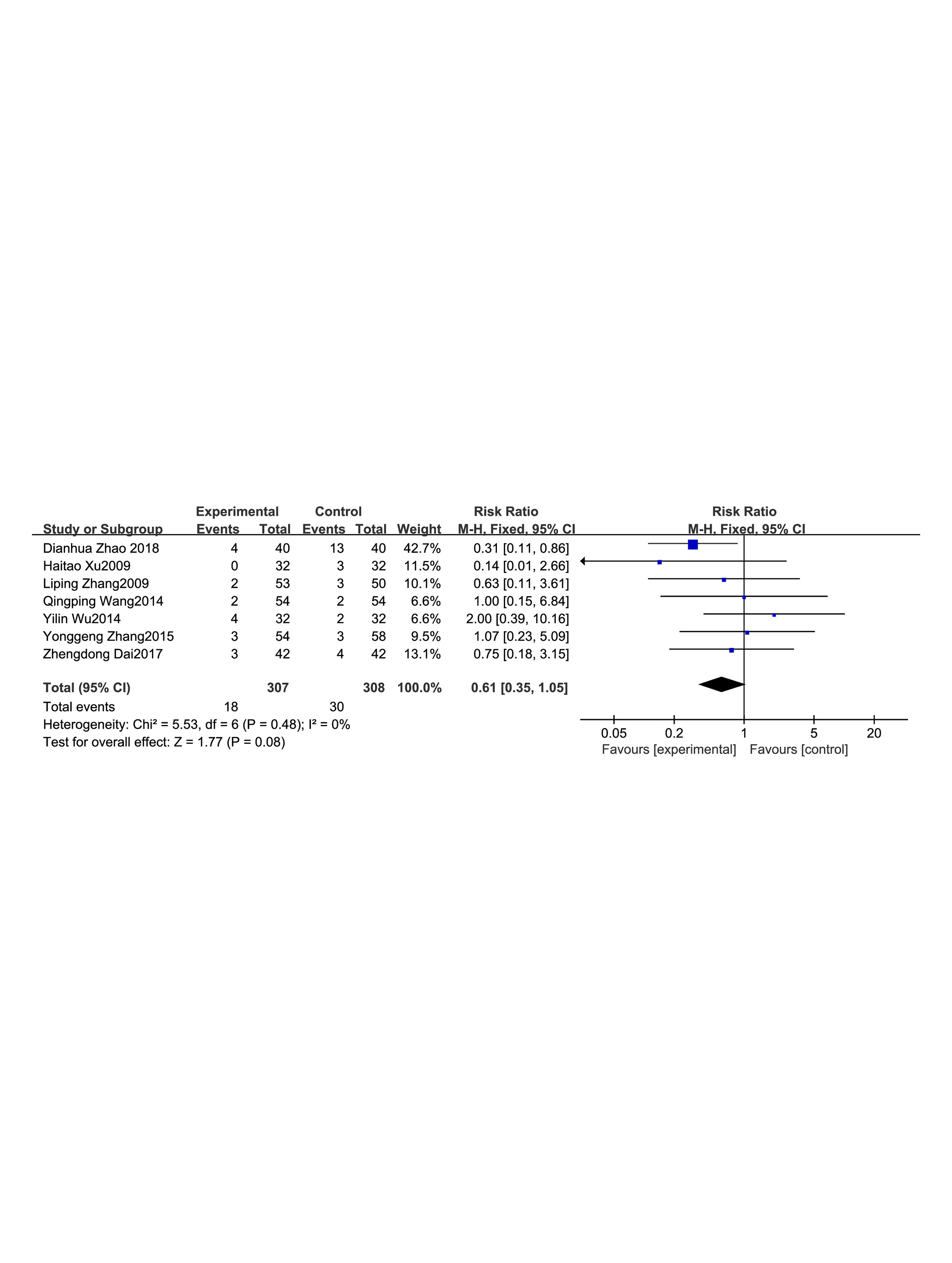

Supplement: Supplementary file 2 [file Data_Sheet_1.zip › Figures/Figure 14 The forest plot of incidence of adverse reactions (pdf.io).tif]

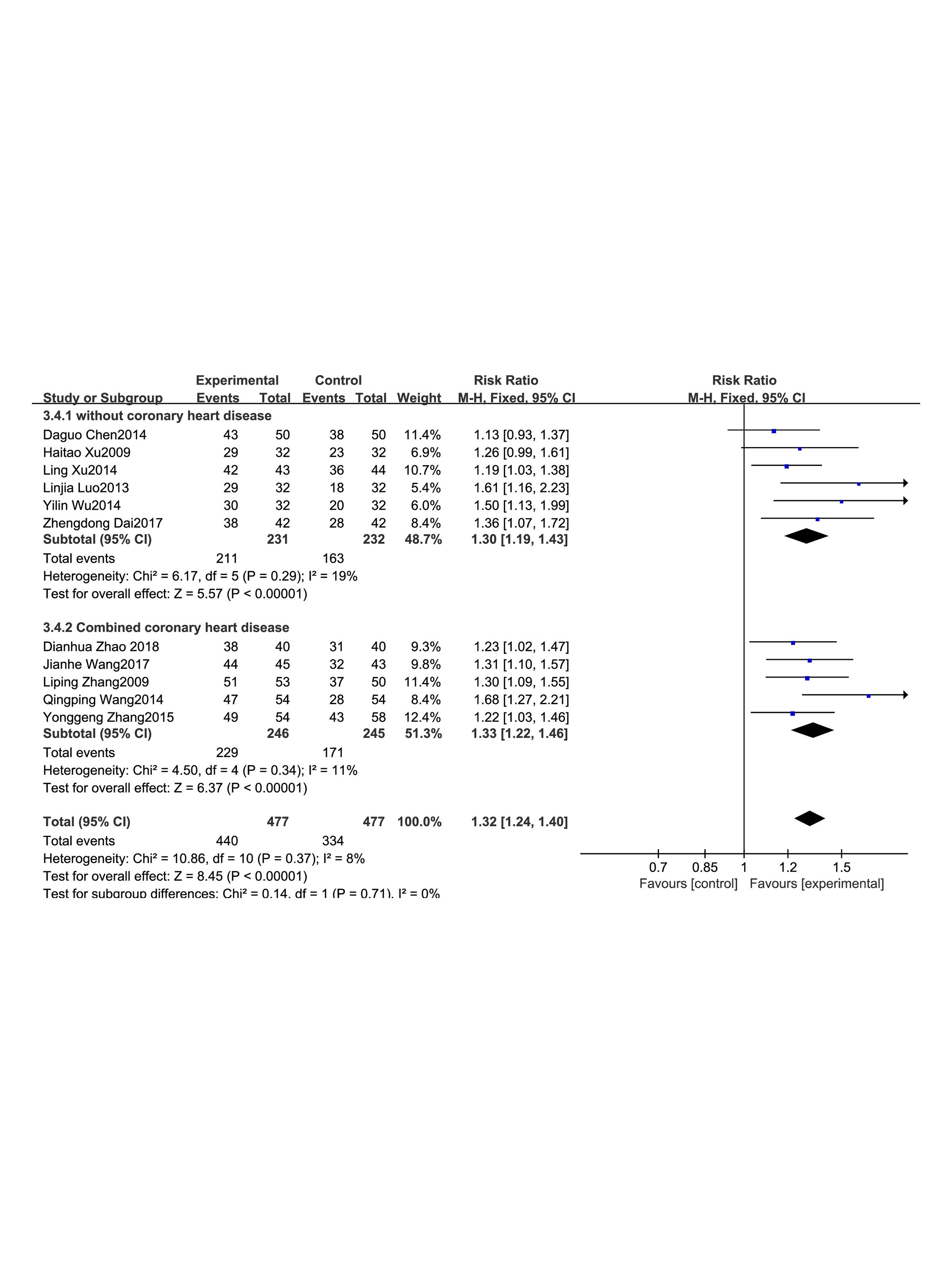

Supplement: Supplementary file 2 [file Data_Sheet_1.zip › Figures/Figure 15 Subgroup analysis of patients with or without coronary heart disease.tif]

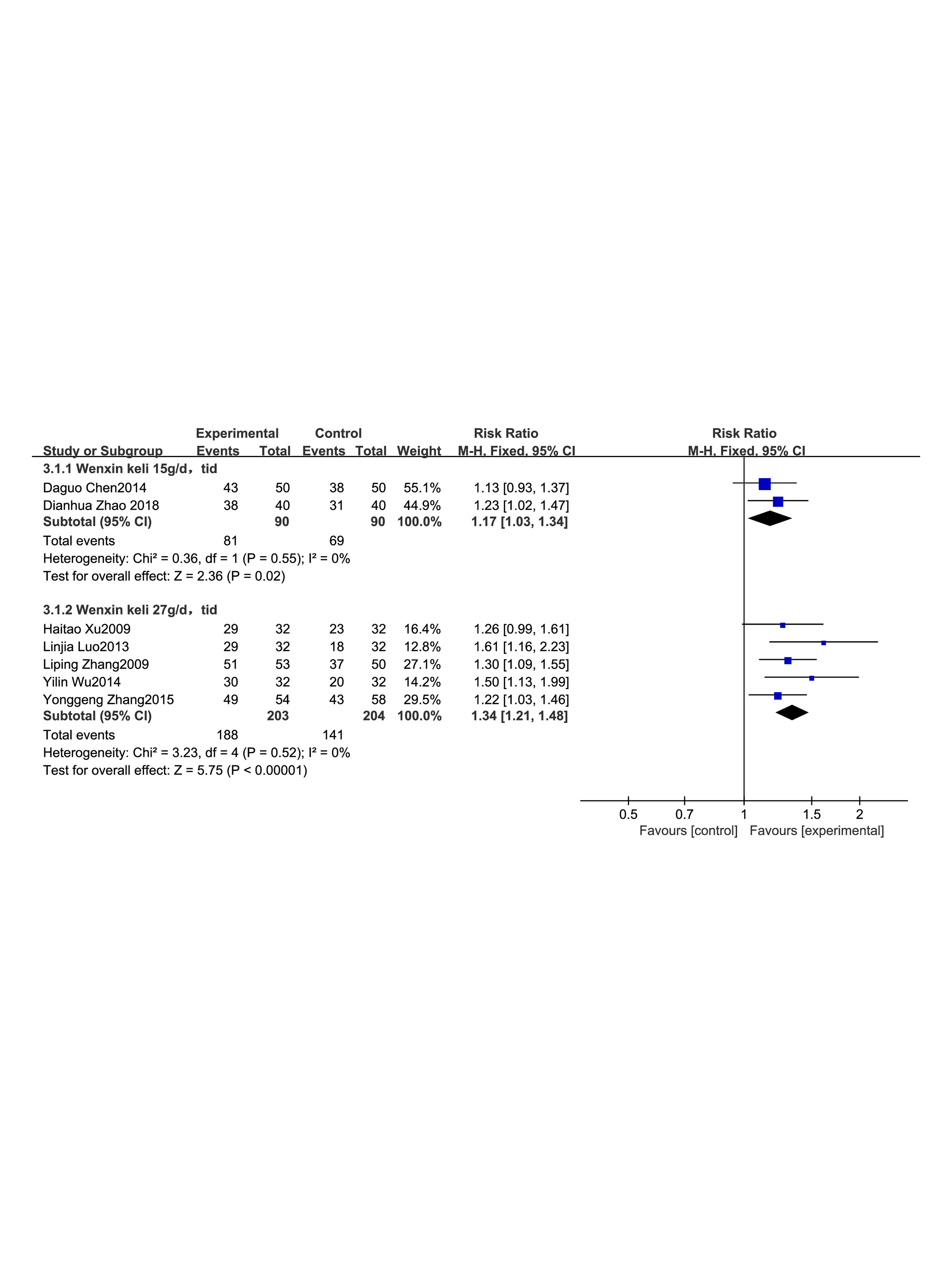

Supplement: Supplementary file 2 [file Data_Sheet_1.zip › Figures/Figure 16 Subgroup analysis of different doses of WXKL.tif]

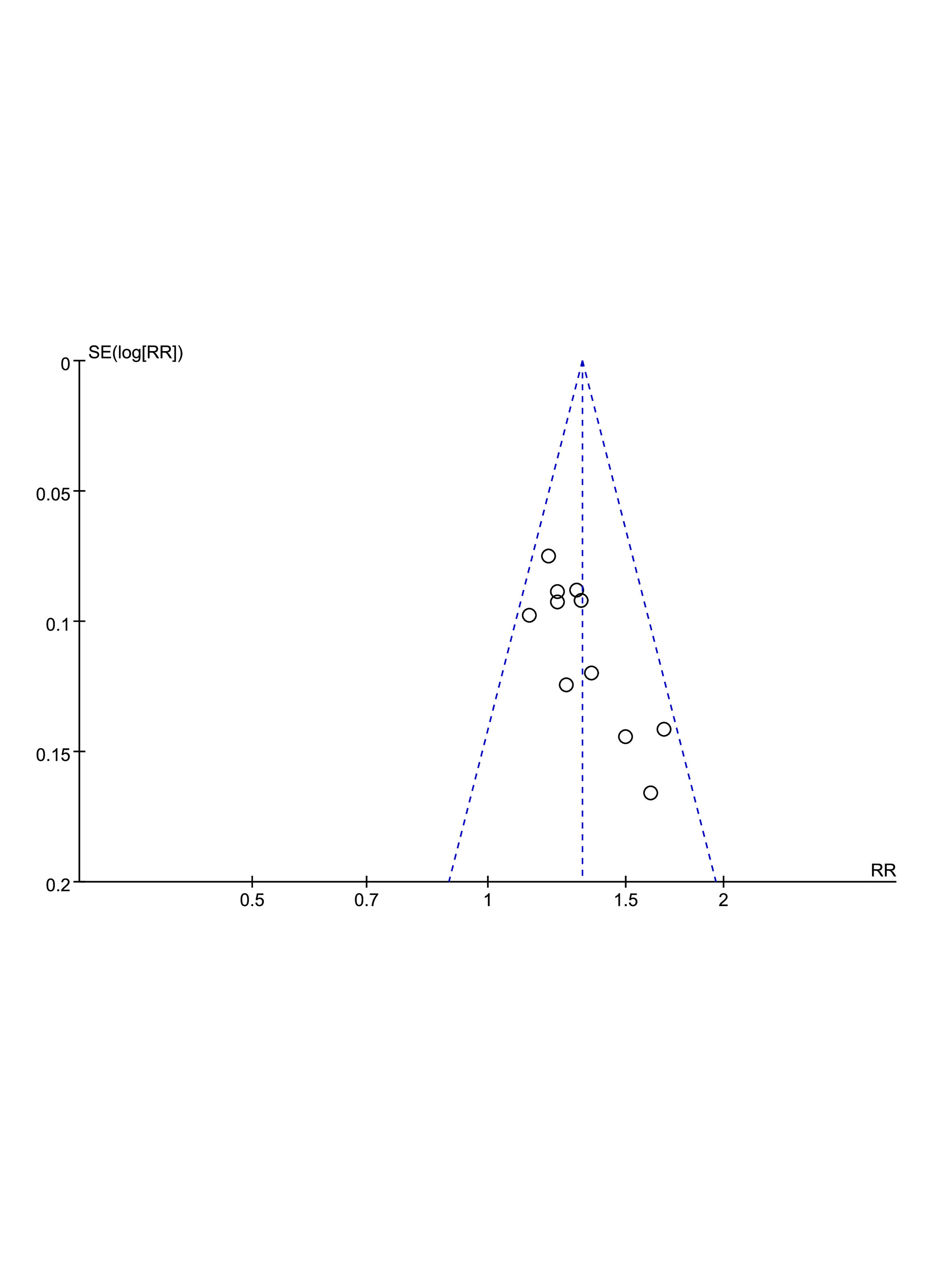

Supplement: Supplementary file 2 [file Data_Sheet_1.zip › Figures/Figure 17 Clinical efficacy funnel plot.tif]

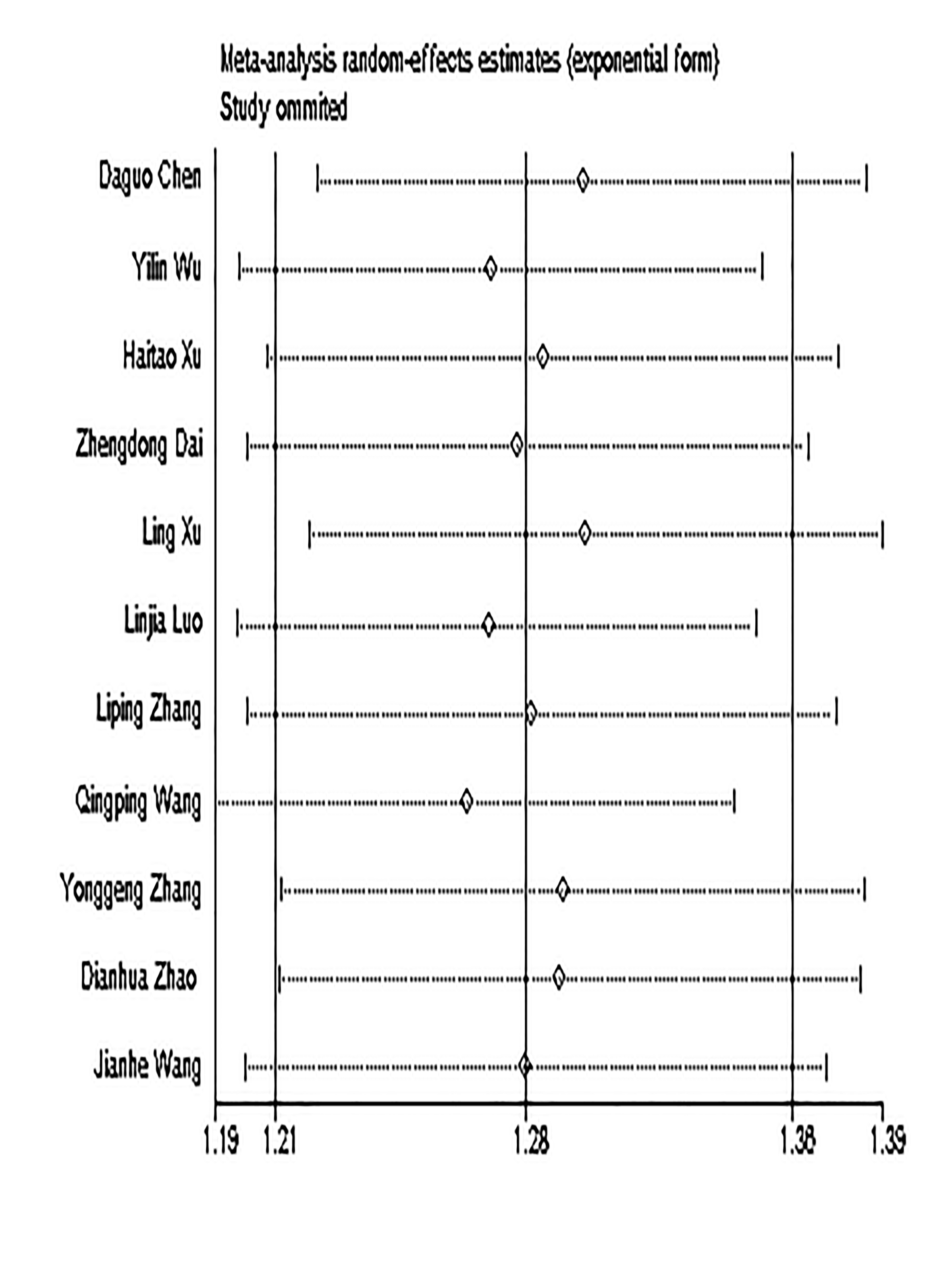

Supplement: Supplementary file 2 [file Data_Sheet_1.zip › Figures/Figure 18 The forest plot of sensitivity analysis.tif]

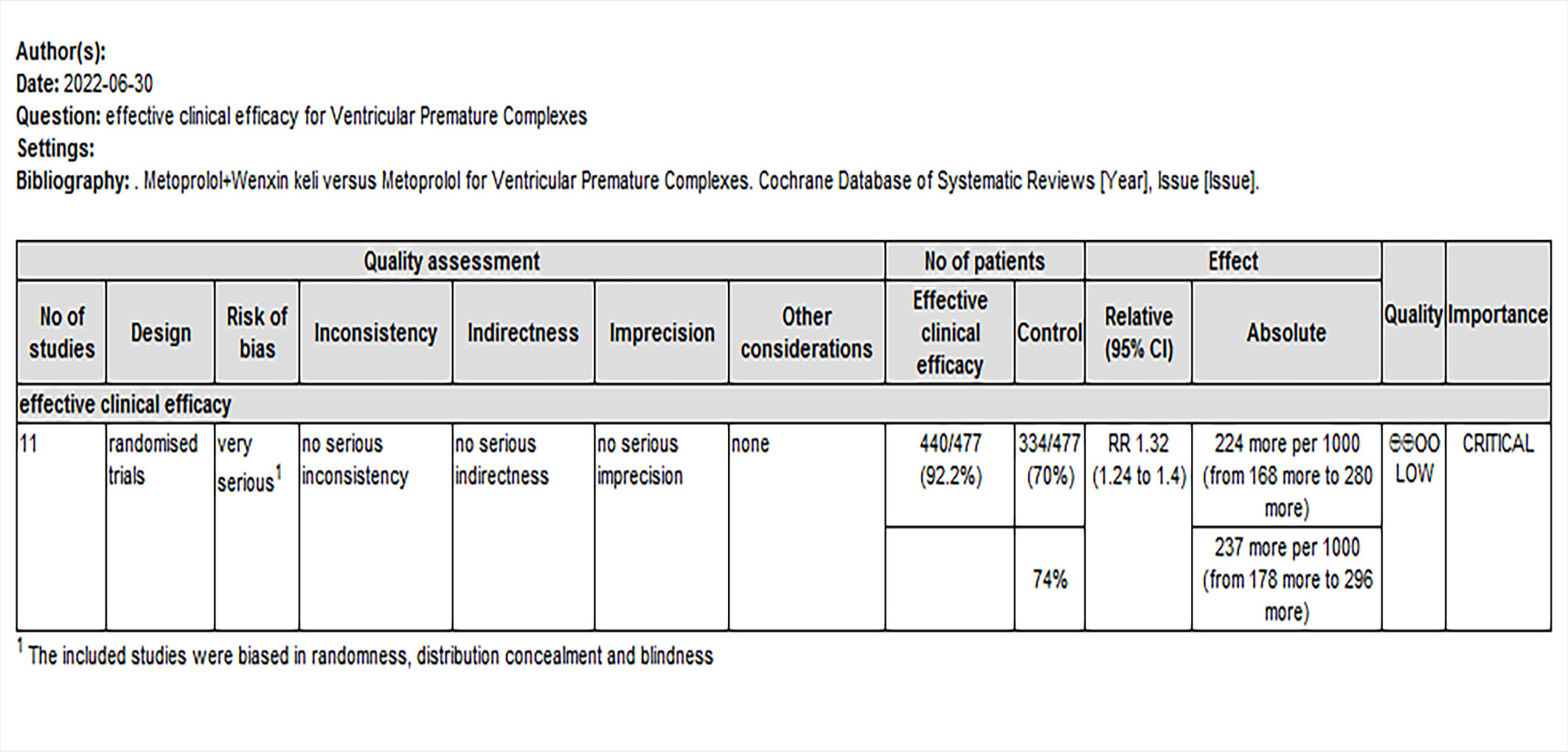

Supplement: Supplementary file 2 [file Data_Sheet_1.zip › Figures/Figure 19 Total effective rate of clinical efficacy.tif]

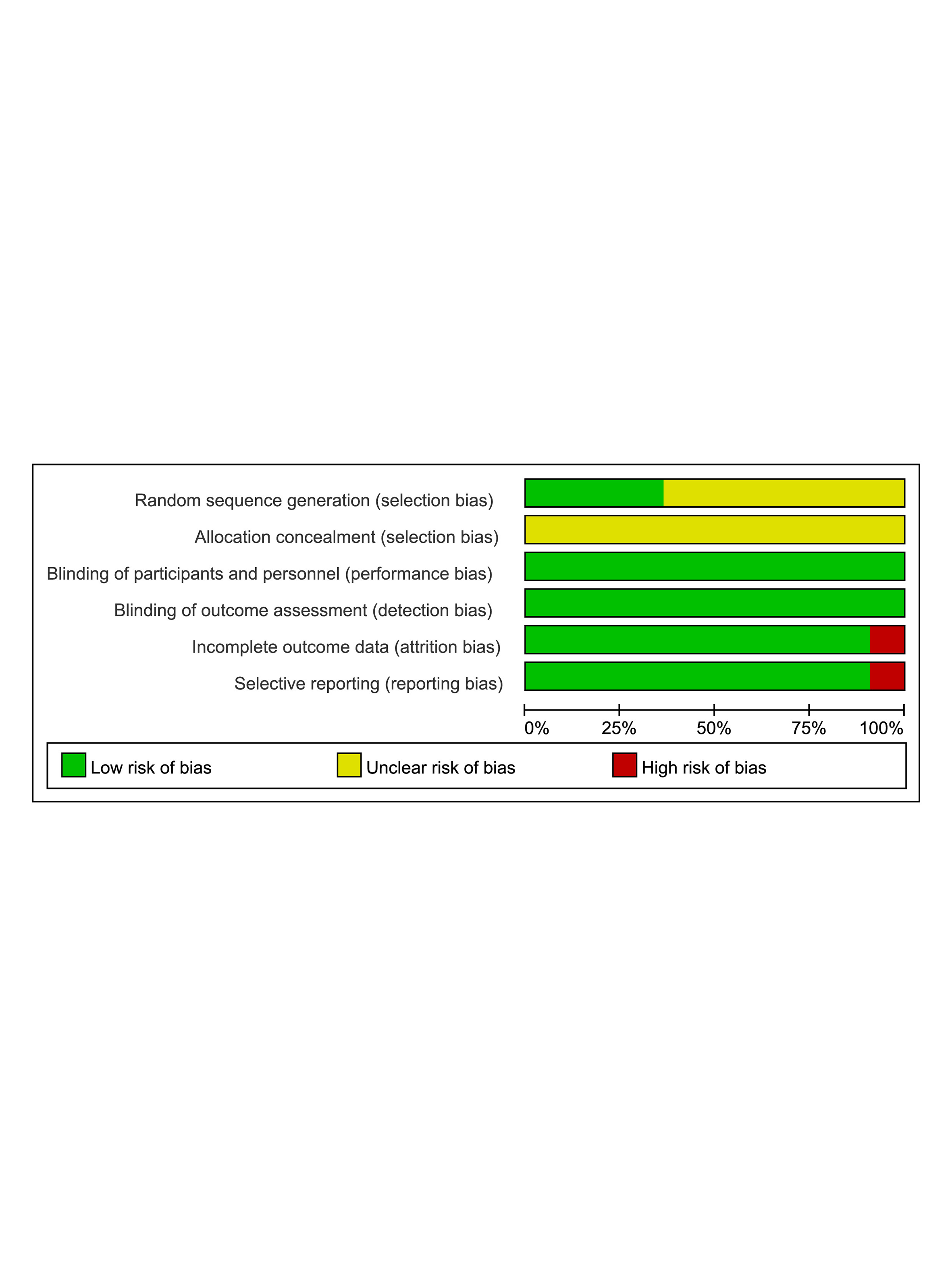

Supplement: Supplementary file 2 [file Data_Sheet_1.zip › Figures/Figure 2 Risk of bias graph.tif]

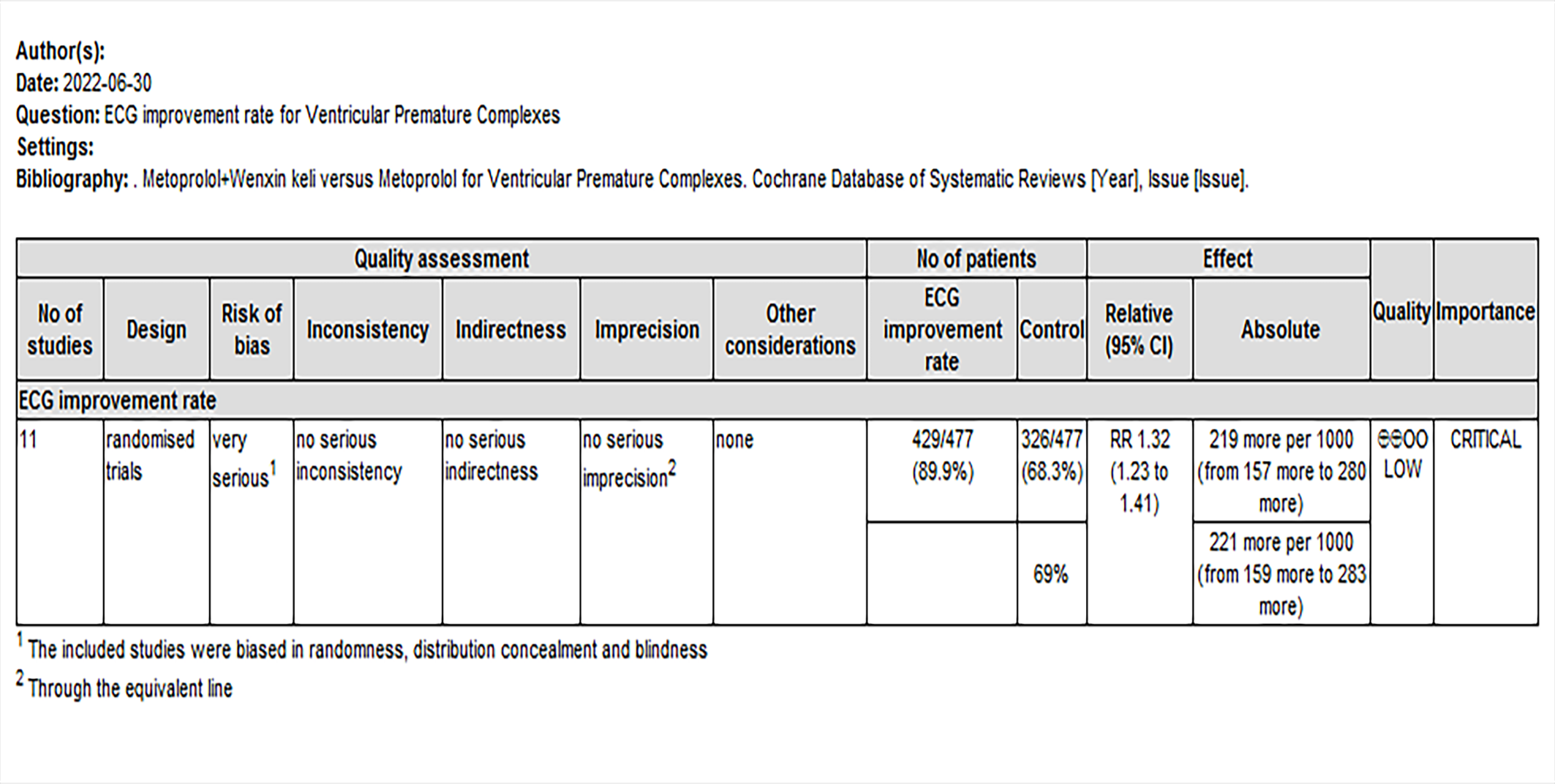

Supplement: Supplementary file 2 [file Data_Sheet_1.zip › Figures/Figure 20 Improvement rate of ventricular premature beat of dynamic electrocardiogram.tif]

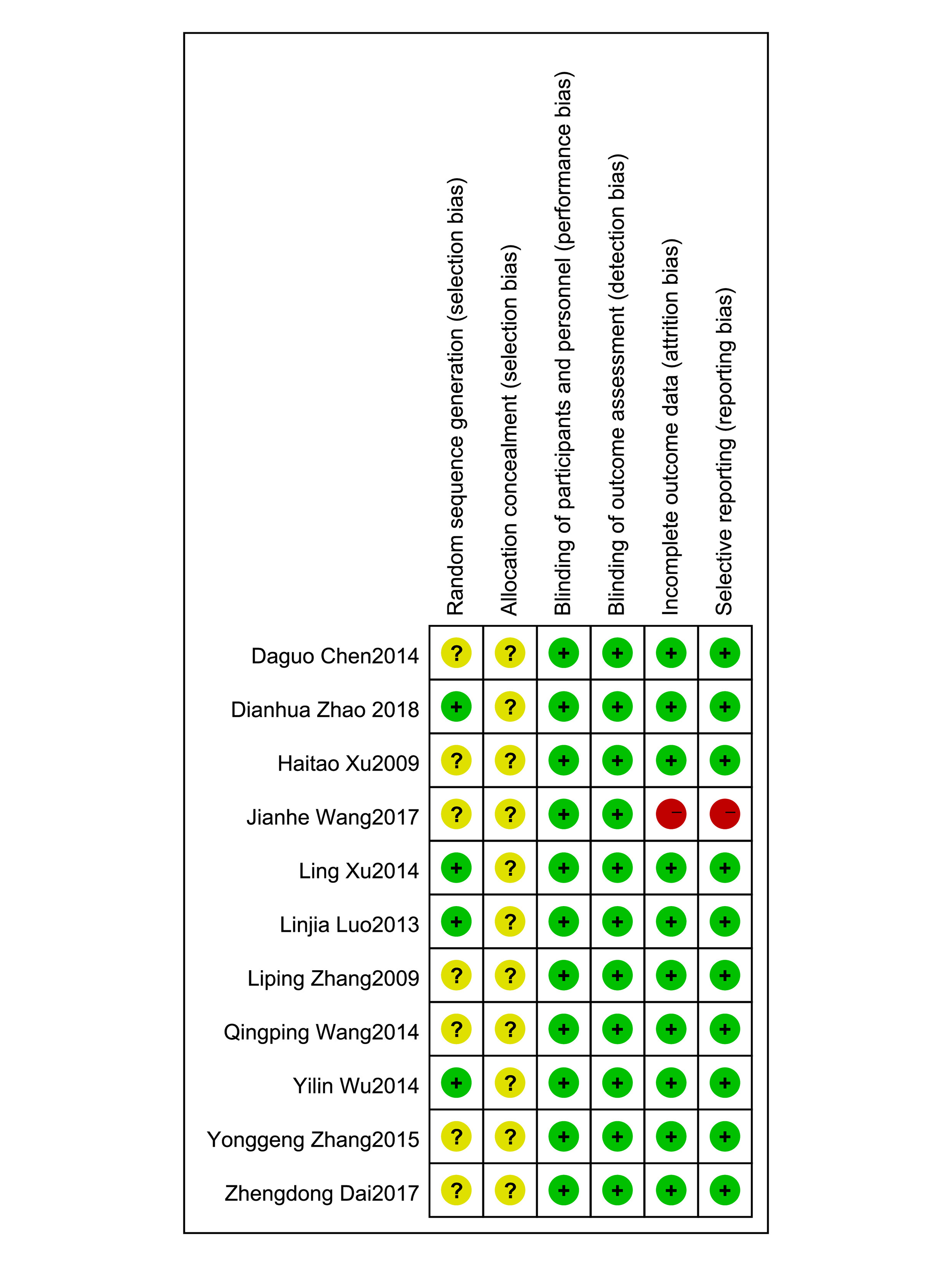

Supplement: Supplementary file 2 [file Data_Sheet_1.zip › Figures/Figure 3 Risk of bias summary.tif]

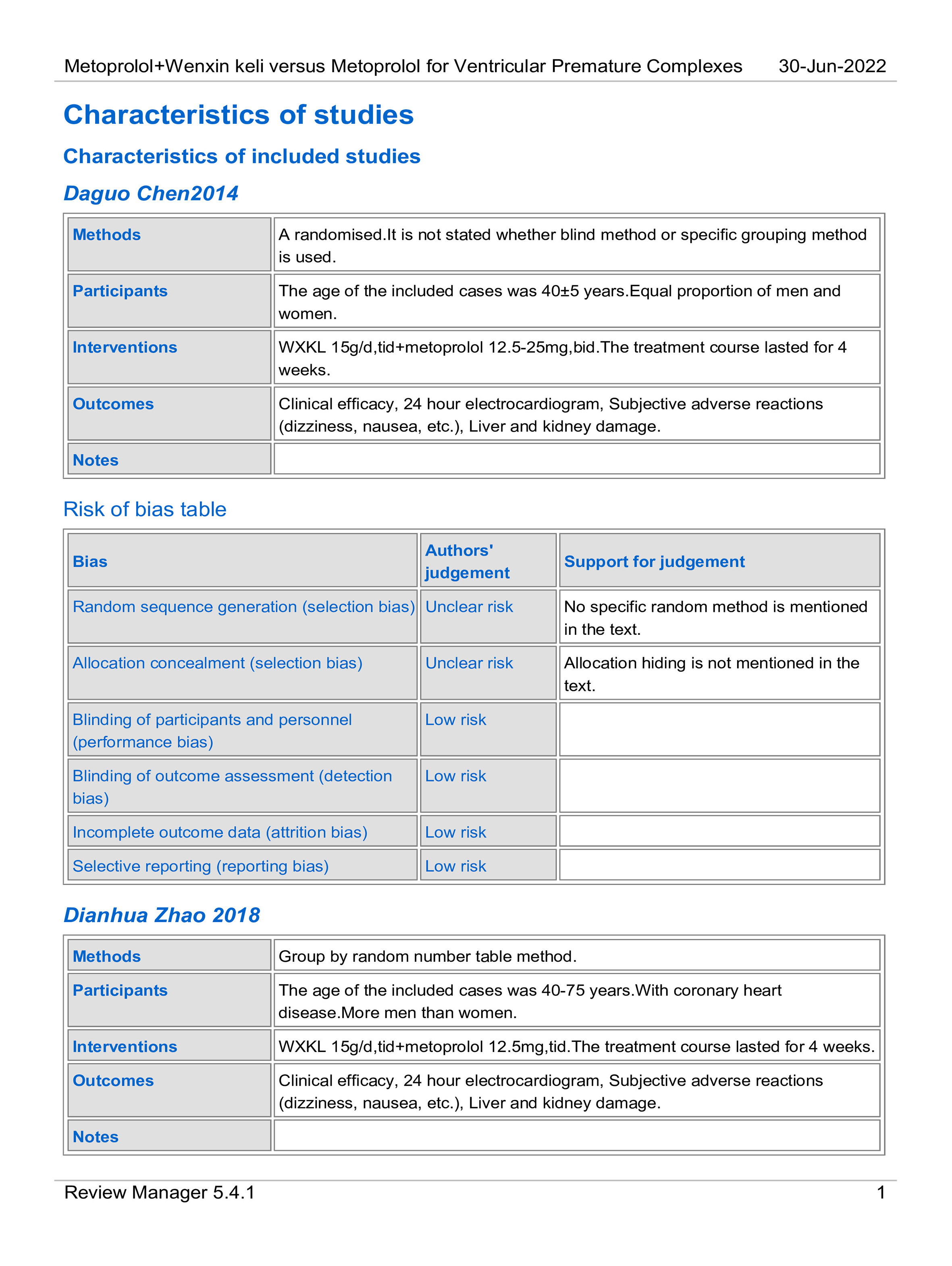

Supplement: Supplementary file 2 [file Data_Sheet_1.zip › Figures/Figure 4 Risk of bias table.tif]

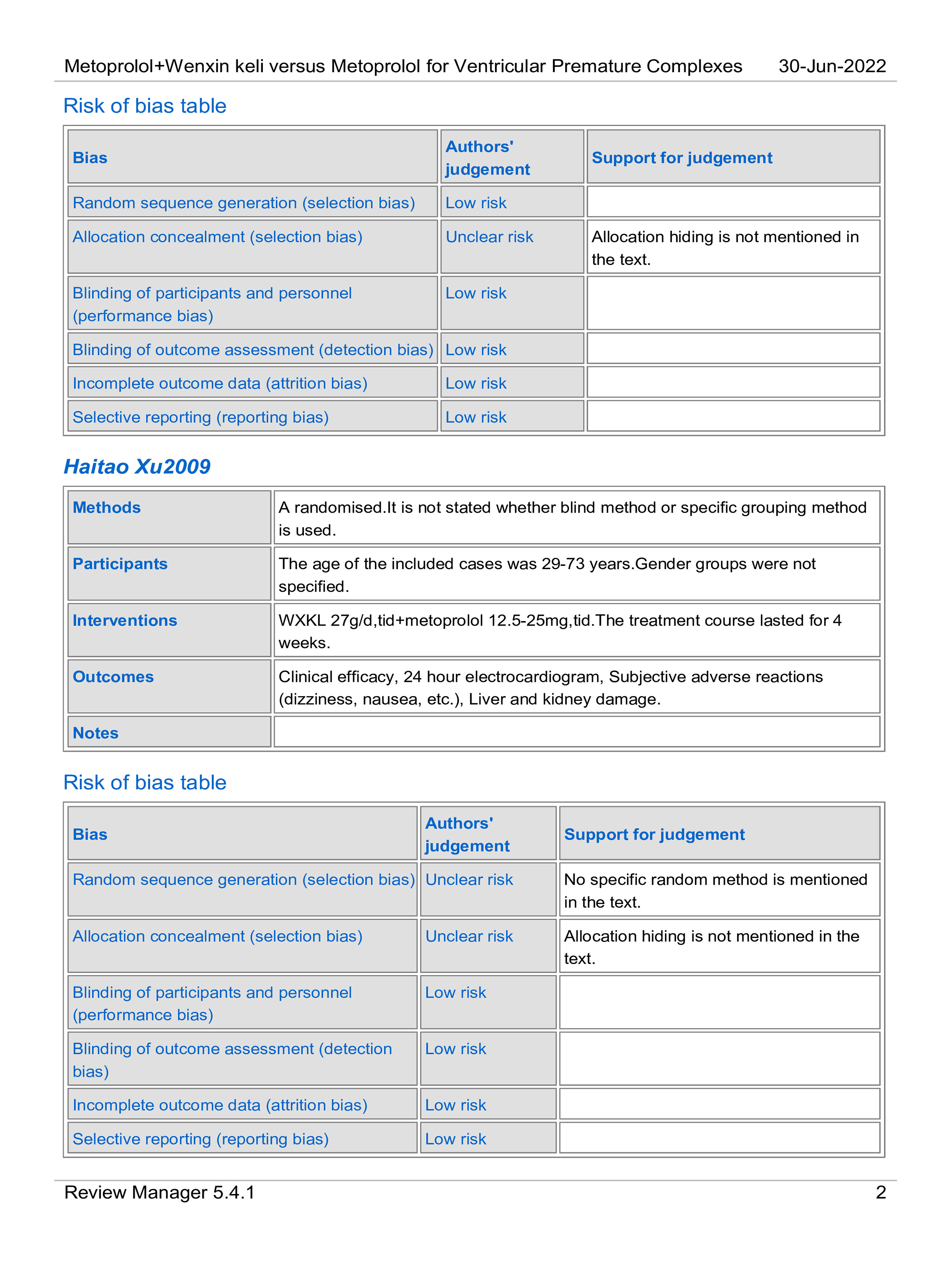

Supplement: Supplementary file 2 [file Data_Sheet_1.zip › Figures/Figure 5 Risk of bias table .tif]

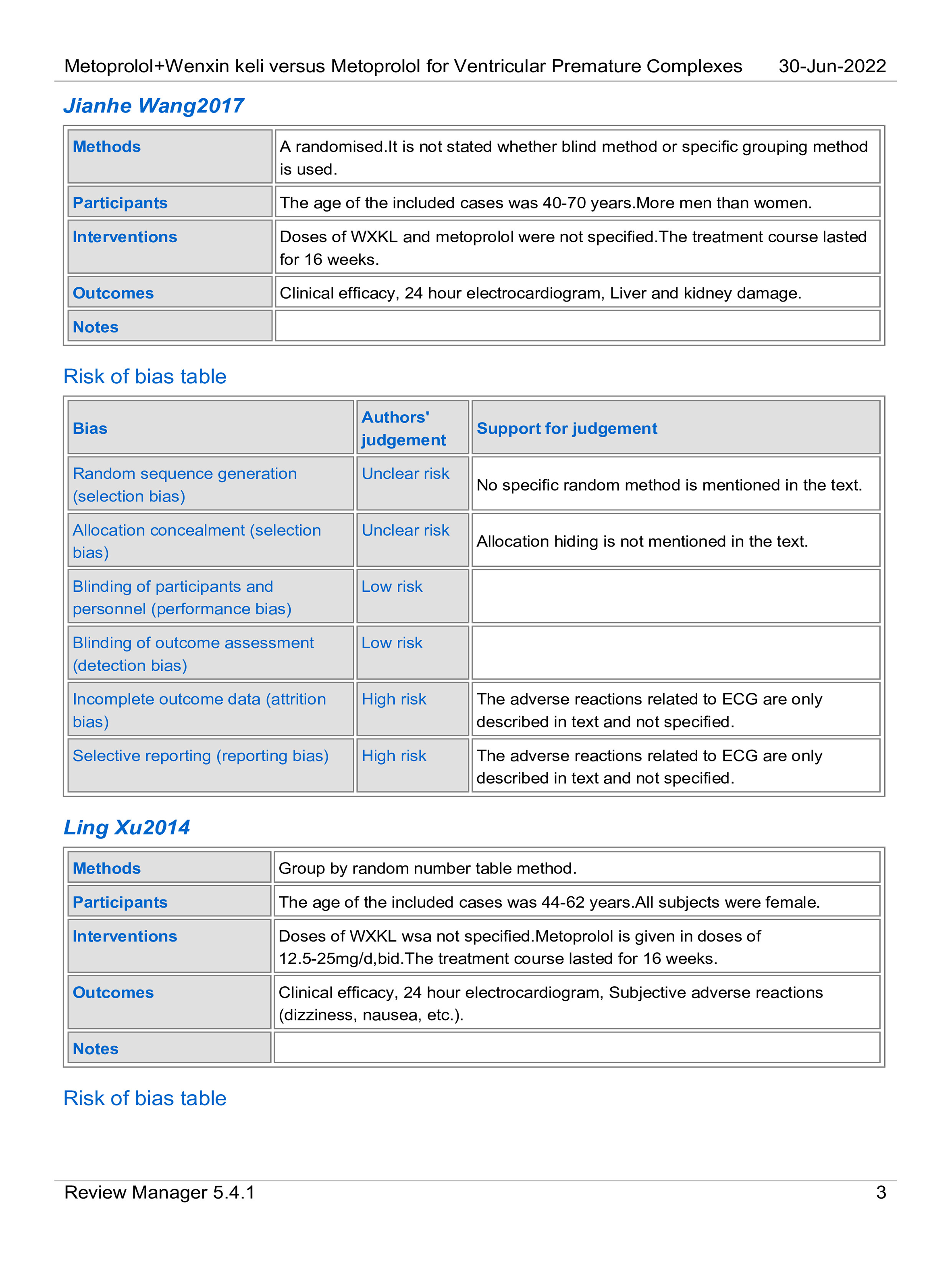

Supplement: Supplementary file 2 [file Data_Sheet_1.zip › Figures/Figure 6 Risk of bias table .tif]

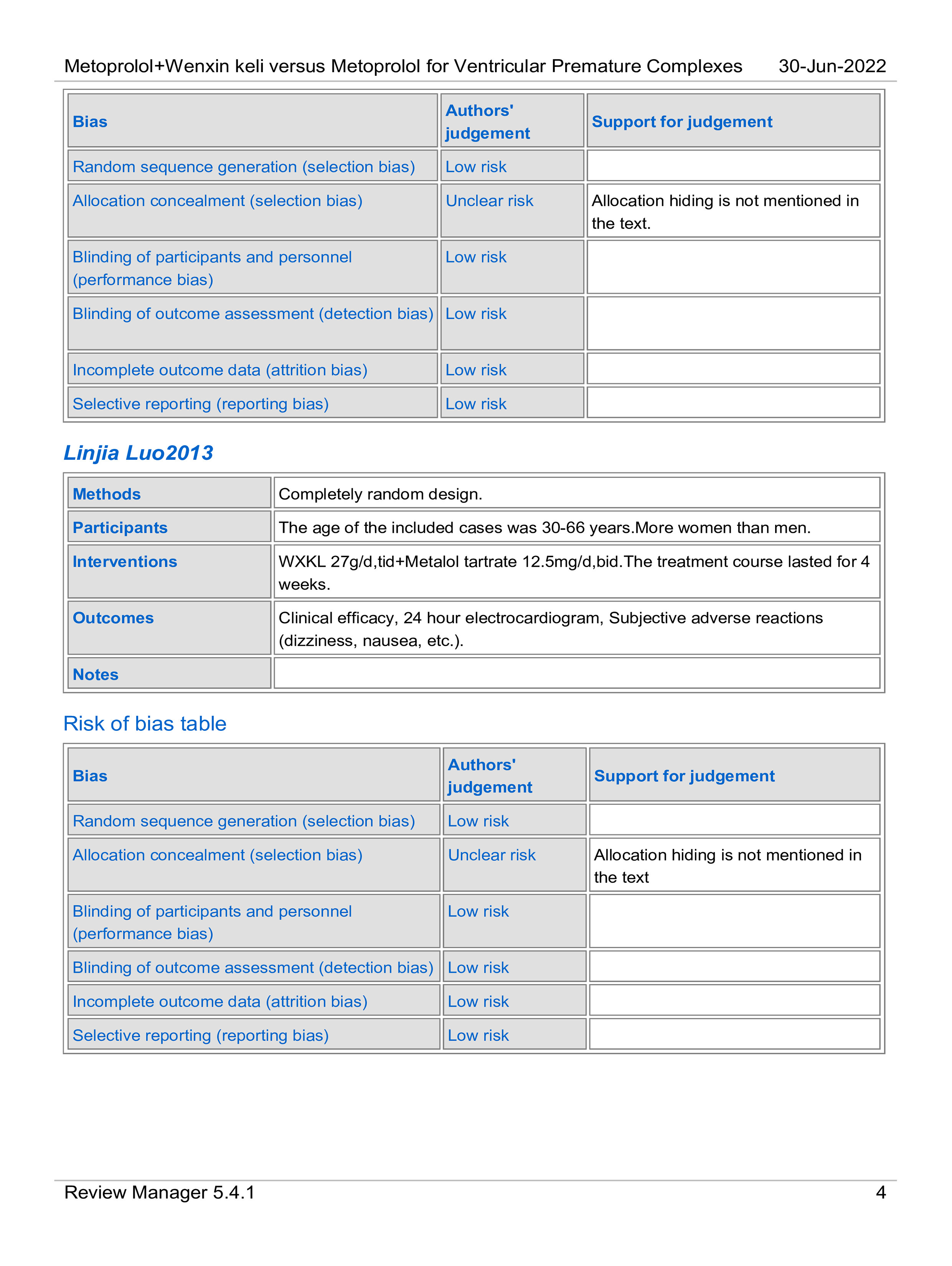

Supplement: Supplementary file 2 [file Data_Sheet_1.zip › Figures/Figure 7 Risk of bias table .tif]

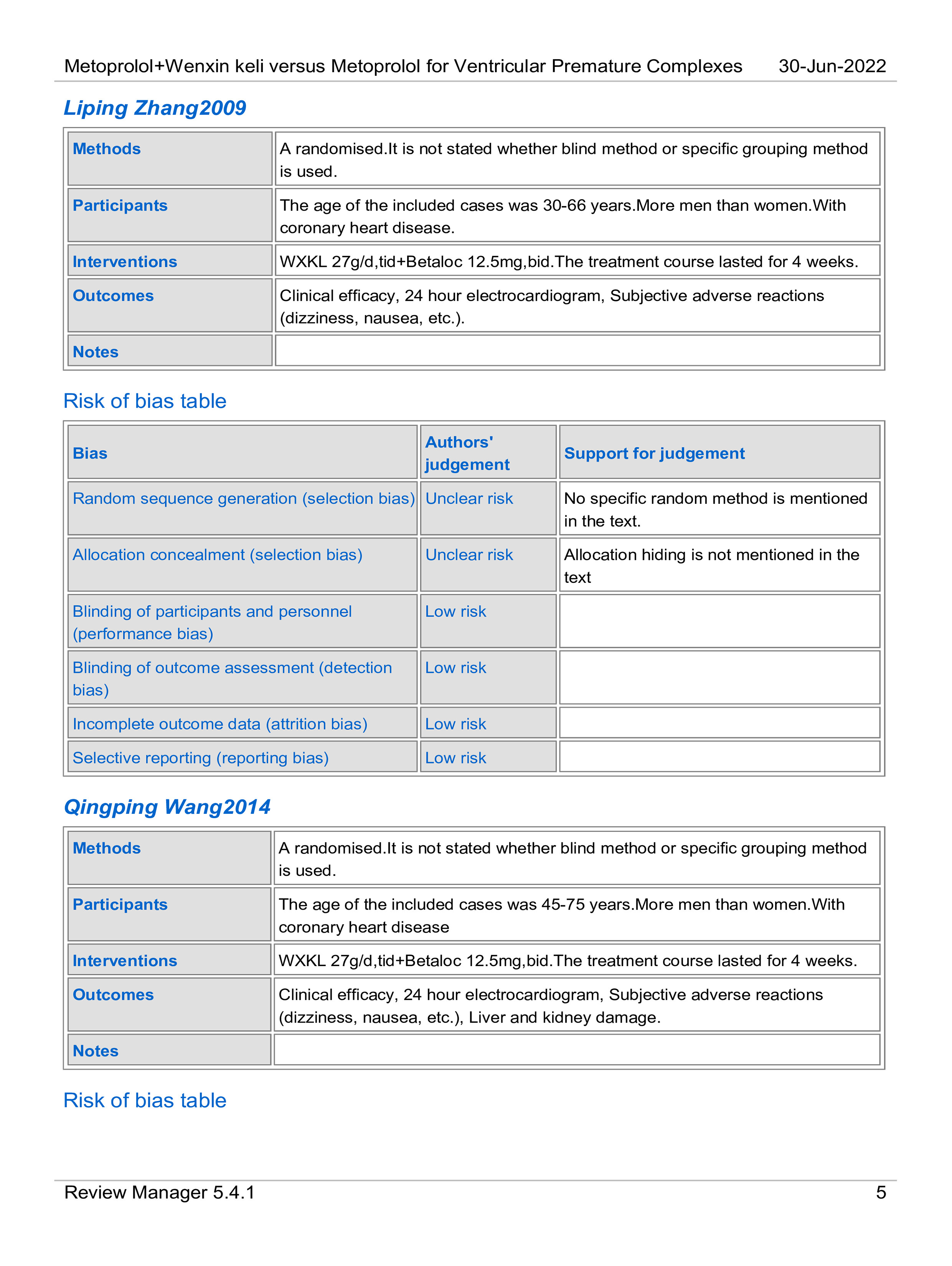

Supplement: Supplementary file 2 [file Data_Sheet_1.zip › Figures/Figure 8 Risk of bias table .tif]

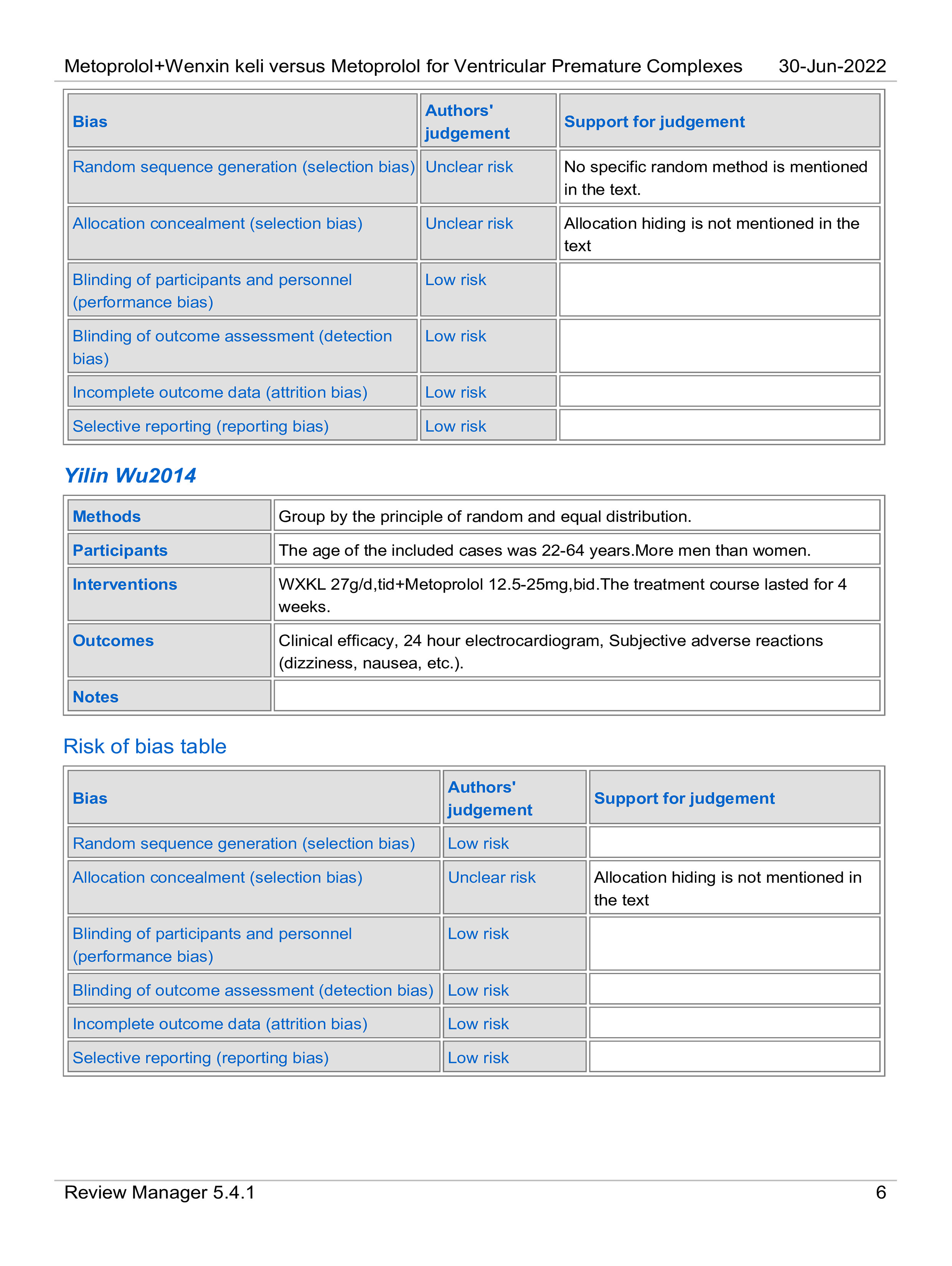

Supplement: Supplementary file 2 [file Data_Sheet_1.zip › Figures/Figure 9 Risk of bias table .tif]

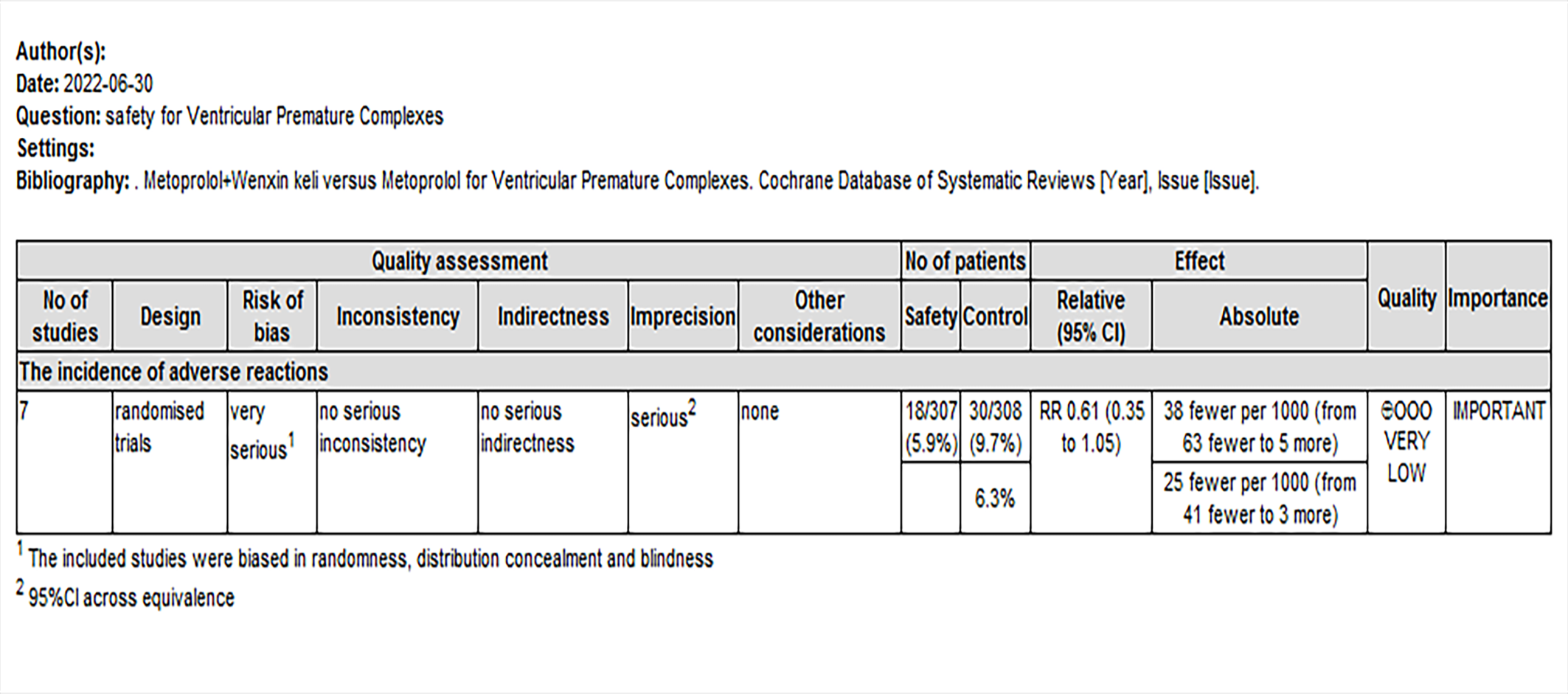

Supplement: Supplementary file 2 [file Data_Sheet_1.zip › Figures/Figure21 Incidence of adverse reactions.tif]
